# One‐Step Construction of Hydrophobic MOFs@COFs Core–Shell Composites for Heterogeneous Selective Catalysis

**DOI:** 10.1002/advs.201802365

**Published:** 2019-02-20

**Authors:** Mengke Cai, Yinle Li, Qinglin Liu, Ziqian Xue, Haiping Wang, Yanan Fan, Kelong Zhu, Zhuofeng Ke, Cheng‐Yong Su, Guangqin Li

**Affiliations:** ^1^ MOE Laboratory of Bioinorganic and Synthetic Chemistry Lehn Institute of Functional Materials School of Chemistry Sun Yat‐Sen University Guangzhou 510275 P. R. China; ^2^ Key Laboratory for Polymeric Composite and Functional Materials of Ministry of Education School of Materials Science and Engineering Sun Yat‐Sen University Guangzhou 510275 P. R. China

**Keywords:** covalent organic frameworks, heterogeneous catalysis, hydrophobic, metal–organic frameworks, selectivity

## Abstract

The exploration of novel porous core–shell materials is of great significance because of their prospectively improved performance and extensive applications in separation, energy conversion, and catalysis. Here, mesoporous metal–organic frameworks (MOFs) NH_2_‐MIL‐101(Fe) as a core generate a shell with mesoporous covalent organic frameworks (COFs) NUT‐COF‐1(NTU) by a covalent linking process, the composite NH_2_‐MIL‐101(Fe)@NTU keeping retentive crystallinity with hierarchical porosity well. Importantly, the NH_2_‐MIL‐101(Fe)@NTU composite shows significantly enhanced catalytic conversion and selectivity during styrene oxidation. It is mainly due to the hydrophilic MOF nanocrystals readily gathering the hydrophobic reactants styrene and boosting the radical mechanism path after combining the hydrophobic COFs shell. The synthetic strategy in this systematic study develops a new rational design for the synthesis of other core–shell MOF/COF‐based hybrid materials, which can expand the promising applications.

## 1 Introduction

The efficient heterogeneous selective catalysis can be envisaged as best happening through a pathway of facile movement of reactants to contact the active sites and the product separated by suitable pore structure as soon as possible.^1^, ^2^, ^3^, ^4^, ^5^ Metal–organic frameworks (MOFs), as a representative porous materials, assembled by metal ions/clusters and functional organic linkers, have inherent porous structures that could be excavated for the development of heterogeneous catalysis.^6^, ^7^, ^8^, ^9^ More than that, the high crystallinity of MOFs, combination with the molecular level design for structural modification, endows the structural optimization to enhance catalytic selectivity.^10^, ^11^ Although the ever‐increasing number of MOFs scaffoldings available for heterogeneous catalysis, their use is primarily hampered by the relatively low stability and easily broken.^12^, ^13^ Furthermore, MOFs are insoluble in most organic solvents and have no precise melting points, which means that MOFs cannot be processed in universal organic solvents or heat treatment techniques.^14^, ^15^ Many substrates of heterogeneous catalysis, such as styrene, are hydrophobic, hence the environmental wettability of catalysts materials also plays a pivotal role in conveying the catalytic performance.^16^, ^17^, ^18^ However, the surface and pore channels of some kinds of MOFs are hydrophilic, such as NH_2_‐MIL‐101(Fe), resulting difficultly gathering hydrophobic substrates. These disadvantages have mostly restrained their practical applications in heterogeneous catalysis.

To overcome this problem, enormous efforts have been made to decorate MOFs with other function materials, thus realizing the association with their advantages and circumventing shortcomings to enhance performance over those of individual ingredients.^19^, ^20^, ^21^, ^22^ A large variety of MOFs composites have been successfully explored, such as MOFs/graphene, MOFs/carbon nanotubes,^23^ MOFs/enzymes,^24^ and MOFs/metal or oxides nanoparticles,^25^, ^26^ integrating the unavailable properties from either component. In addition, porous covalent organic frameworks (COFs), a kind of emerging porous crystalline materials thoroughly formed by organic molecules used covalent bonds process, are also attracting extensively research attention especially in catalysis, gas storage, separations, and drug delivery.^27^, ^28^, ^29^, ^30^ Very recently, the hybrid core–shell MOFs@COFs composites, such as NH_2_‐MIL‐68@TPA‐COF and NH_2_‐UiO‐66/TpPa‐1‐COF, have been synthesized and presented efficient in photocatalytic dye degradation and photocatalytic H_2_ evolution, respectively.^31^, ^32^, ^33^ However, the novel core–shell MOFs@COFs composites for heterogeneous catalysis still remains largely unexplored, and it is also difficult to adjust the hydrophilic‐hydrophobic properties through linking hydrophobic COFs shell to the hydrophilic MOFs nanocrystals. In this work, we aim to develop stable core–shell MOFs@COFs composites by a one‐step modification, which enables the crystalline and hierarchical porosity materials and adjustable hydrophilic‐hydrophobic properties for efficient heterogeneous selective catalysis.

## 2 Results and Discussion

**Scheme 1:**
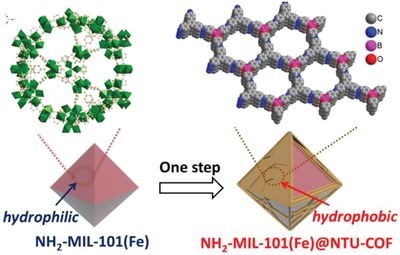
Fabrication of NH_2_‐MIL‐101(Fe)@NTU‐COF.

Here, we demonstrate a facile method to fabricate a new type of MOFs@COFs core–shell hybrid materials (**Scheme** **1**). NH_2_‐MIL‐101(Fe) as a MOF has been employed to set as core because of its high surface area, stubborn stability, and flexible modification.^34^, ^35^ By one‐step synthesis, for the first time, a two‐component covalent organic frameworks (COFs) NUT‐COF‐1 (NTU‐COF) was controlled to grow on NH_2_‐MIL‐101(Fe) by the formations of imine group and boroxine ring, involving the utilization of two building blocks of 4‐formylphenylboronic acid (FPBA) and 1,3,5‐tris(4‐aminophenyl)‐benzene (TAPB).^36^, ^37^ Both NH_2_‐MIL‐101(Fe) and NTU‐COF show relatively crystalline X‐ray diffractions patterns as synthesized, consistent with the simulated results (see Figures S1 and S2 in the Supporting Information). The condensation reaction between 2‐aminoterephthalic acid and FPBA results in covalent anchoring of FPBA to the surface of NH_2_‐MIL‐101(Fe). Then unreacted –B(OH)_2_ groups can be used as nucleation sites for NTU‐COF. With the variation of amounts of FPBA and TAPB, a series of NH_2_‐MIL‐101(Fe)@NTU‐COF samples were synthesized, shown in optical images (Figure S3, Supporting Information) and named as MIL@NTU‐*x*, with the mass ratio of *x* = 1, 2, 3, 4, or 5.

**Figure 1:**
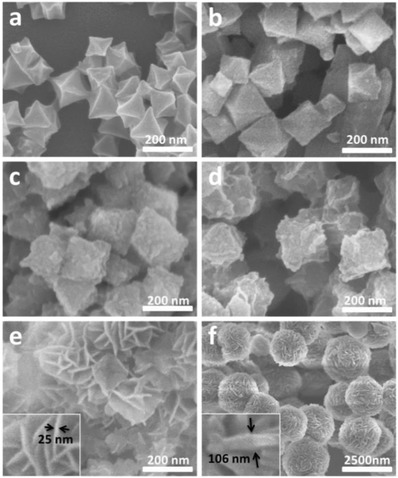
FESEM images of a) NH_2_‐MIL‐101(Fe), b) MIL@NTU‐1, c) MIL@NTU‐2, d) MIL@NTU‐3, e) MIL@NTU‐4, and f) NTU‐COF. The insets in (e,f) show the corresponding local magnification nanosheets.

The morphology of the as‐synthesized NH_2_‐MIL‐101(Fe) is characterized by field‐emission scanning electron microscopy (FESEM) and transmission electron microscopy (TEM). The FESEM image (**Figure** **1**a) and TEM image (Figure S4, Supporting Information) indicate that the obtained NH_2_‐MIL‐101(Fe) is uniform octahedrons with smooth surface and a diameter of about 200 nm. After coating, the amounts of coated shell were found to be a huge impact for formation of nanostructures with varied morphology. As shown in Figure 1b–d, the typical samples MIL@NTU‐*x* (*x* = 1–3), coating with NTU, still inherited the similar octahedral morphology of the NH_2_‐MIL‐101(Fe) but presented obvious rougher surface. The TEM images (Figures S5 and S6, Supporting Information) clearly elucidate the core–shell nature of the MIL@NTU‐*x* (*x* = 2 and 3) by the sharp contrast between the (20–40 nm) sheet‐like shell and the central core. The shell thickness of MIL@NTU‐1 could not be estimated from TEM image (Figure S7, Supporting Information) because of too thin and less than 20 nm considering the amount of starting materials for construction of NTU‐COF. Interestingly, it is noteworthy that the NTU‐COF grown on the surface of NH_2_‐MIL‐101(Fe) exhibited the unique structure feature. With high coating amount (*x* = 4 and 5), individual nanostructures began to adhere to each other (Figures S8 and S9, Supporting Information) and the NTU‐COF nanosheets were found to be vertically standing on the surface of the hybrid materials (Figure 1e and Figure S10: Supporting Information). The thicknesses of NTU‐COF nanosheets for MIL@NTU‐4 and MIL@NTU‐5 were the same (Figure 1e and Figure S10: Supporting Information insets; both nanosheets thickness were 25 nm). While the individual NTU‐COF is composed of uniform hollow microspheres (Figure 1f and Figure S11: Supporting Information), and the microspheres consist of the random nanosheets with the thickness of 106 nm (Figure 1f inset). Furthermore, it is the first time that highly ordered COFs nanosheets can be achieved by stepwise core–shell coating on MOFs, suggesting a strategy to control the morphology of COFs.

**Figure 2:**
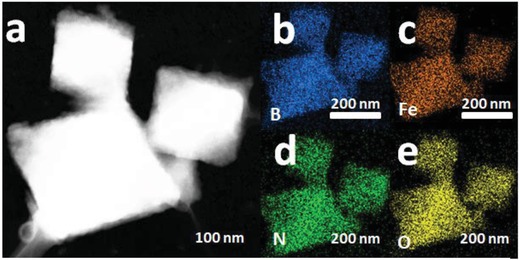
a) HAADF‐STEM image of MIL@NTU‐1. Elemental mapping images of b) B, c) Fe, d) N, and e) O of the structure shown in (a).

To obtain more direct evidence, we used energy dispersive X‐ray spectroscopy (EDX) to map the distribution of different elements in MIL@NTU‐1 using high‐angle annular dark field scanning transmission electron microscopy (HAADF‐STEM) (**Figure** **2**a). The elemental mapping (Figure 2b–e) showed that B, Fe, N, and O elements distributed homogeneously throughout individual nanostructure. The accurate distribution of elements in single hybrid structure was further analyzed by EDX line scan. In Figure S12 in the Supporting Information, N, O, and Fe elements became richer gradually from surface to interior, while the Fe element only from NH_2_‐MIL‐101(Fe) was not detected at the edge of the particle. These results index that the core–shell structured material was formed and the NTU shell was very thin about 6–11 nm, which coincided well with the TEM results. As a contrast, the elemental mapping of MIL@NTU‐3 clearly showed that NH_2_‐MIL‐101(Fe) was covered with NTU (Figure S13a–d, Supporting Information). With the aforementioned works, it can be concluded that the amounts of FPBA and TAPB provide a key role to control the thickness of shell and morphology for constructing MOFs@COFs core–shell hybrid material.

**Figure 3:**
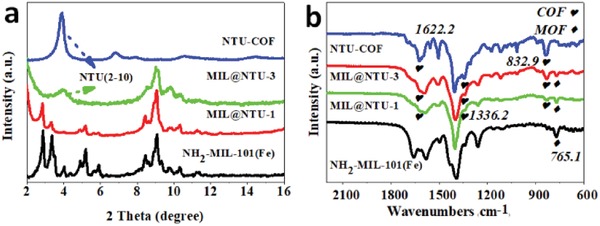
a) XRD patterns of NH_2_‐MIL‐101(Fe), MIL@NTU‐1, MIL@NTU‐3, and NTU‐COF. b) FT‐IR spectra of NTU‐COF, MIL@NTU‐1, MIL@NTU‐3, and NH_2_‐MIL‐101(Fe).

**Figure 4:**
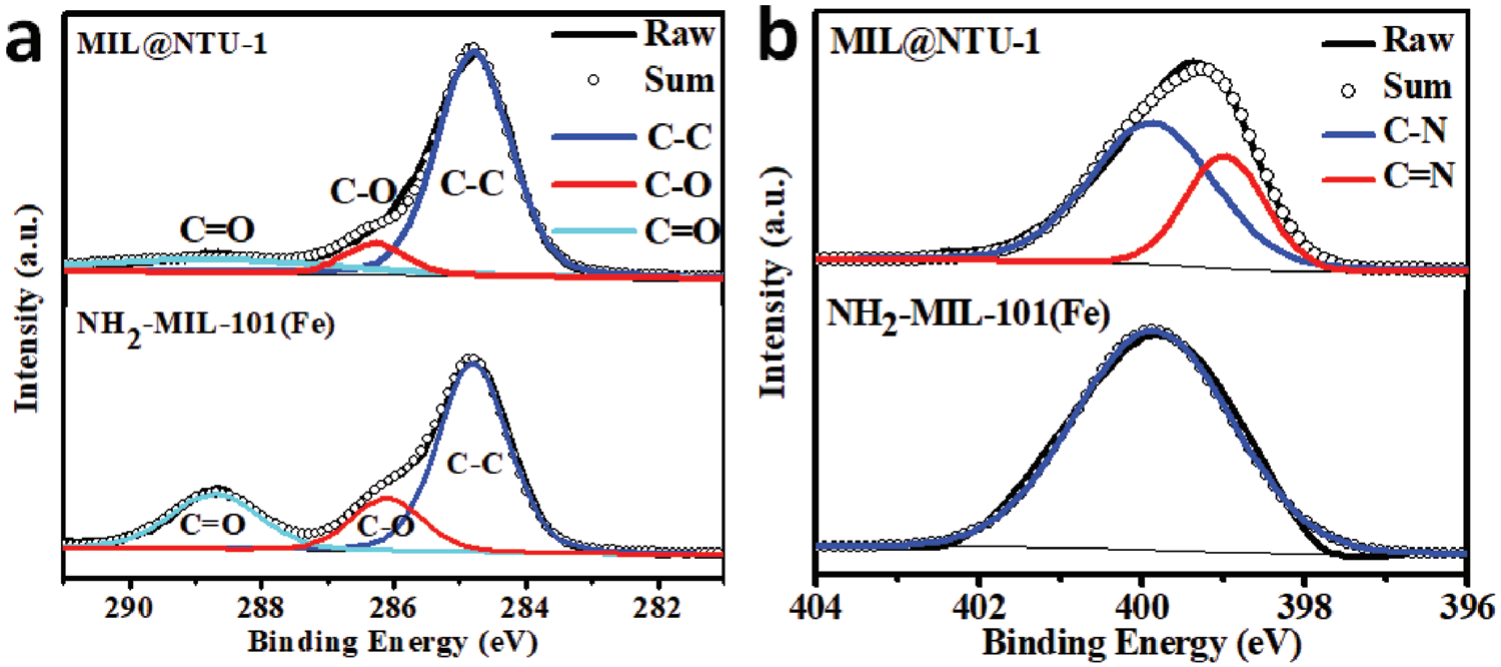
a) C 1s and b) N 1s XPS of NH_2_‐MIL‐101(Fe) and MIL@NTU‐1.

The X‐ray powder diffraction (XRD) was further carried out to verify the NTU‐COF formation on the surface of NH_2_‐MIL‐101(Fe). As shown in **Figure** **3**a, MIL@NTU‐1 showed no obvious diffraction peaks of NTU‐COF perhaps due to low coating NTU‐COF. With more coating, the presence of a new characteristic diffraction peak at 4.2°, originating from the (2–10) plane of NTU‐COF, confirmed the crystallinity of the MIL@NTU‐3. Corresponding to the shell thickened, the characteristic diffraction peak of NTU‐COF could be observed for the MIL@NTU‐*x* (*x* = 4 and 5) but not obvious for the MIL@NTU‐2 (Figure S13, Supporting Information), ascribed to that NTU‐COF possess relatively poor crystallinity versus MOFs and the shell is relatively thin (<20 nm). Moreover, the thicker shell, the poorer reflections of NH_2_‐MIL‐101(Fe) (Figure 3a and Figure S14: Supporting Information), indicating that the surface coating caused the collapse of the core structure. The Fourier transform infrared (FT‐IR) spectrum of the core–shell structure matches well with NH_2_‐MIL‐101(Fe) and NTU‐COF (Figure 3b and Figure S15: Supporting Information). The new characteristic peaks at 832.9, 1336.2 (B—O), and 1622.2 cm^−1^ (C=N) originating from the shell, indicated the formation of NTU‐COF.^38^ Furthermore, the electronic state of element in MIL@NTU‐*x* was studied by the X‐ray photoelectron spectroscopy (XPS) analyses. XPS signal intensity is affected by penetration depth of the X‐rays used in XPS,^39^ which can be used as a method to study the shell thickness in encapsulation process. From **Figure** **4**a and Figures S16 and S17 (Supporting Information), the C 1s peak can be deconvoluted into three peaks ascribed to C—C (284.8 eV), C=O (286.1 eV), and C—O (288.7 eV) groups, revealing the dramatically decreased –COOH functional group after NTU‐COF coating. Meanwhile, the N 1s peak can be deconvoluted into three peaks ascribed to C‐N (284.8 eV) and C=N (286.1 eV) groups (Figure 4b and Figure S18: Supporting Information), revealing the dramatically decreased C=N functional group after NTU‐COF coating. Both are consistent with that the NTU‐COF contains no –COOH but C=N functional group compared with NH_2_‐MIL‐101(Fe). In addition, the peak area of B 1s from NTU‐COF was obviously increasing with shell thickened, but decreased for Fe 2p from NH_2_‐MIL‐101(Fe), further confirming that constructing core–shell structure NH_2_‐MIL‐101(Fe)@NTU‐COF was succeeded (Figures S19 and S20 and Table S1, Supporting Information). The thermogravimetric analysis (TGA) proved that the MIL@NTU‐*x* core–shell material inherited the high thermal stability of NTU‐COF up to 400 °C instead of 200 °C for NH2‐MIL‐101(Fe) (Figure S21, Supporting Information). Additionally, the original morphology of MIL@NTU‐3 can be preserved even after carbonization (see details in Figures S22 and S23 in the Supporting Information). In contrast, NH_2_‐MIL‐101(Fe) without NTU‐COF coated was easily collapsed during heat treatment (Figures S24 and S25, Supporting Information).

**Figure 5:**
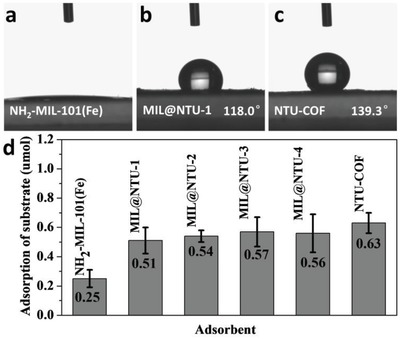
Water contact angle measurements of a) NH_2_‐MIL‐101(Fe), b) MIL@NTU‐1, and c) NTU‐COF. d) Styrene adsorption (see details in the Supporting Information) with 10 mg materials.

Hydrophilicity/hydrophobicity plays a very important role in catalysis for nanomaterials.^40^ To further probe the surface properties of the MIL@NTU‐*x*, the water contact angle was measured on a pellet of each material. NH_2_‐MIL‐101(Fe) showed complete water wetting (**Figure** **5**a), which is consistent with previous report.^25^ The water contact angles gradually increased from 118.0° (MIL@NTU‐1) to 137.1° (MIL@NTU‐4) shown in Figure 5b and Figure S26: Supporting Information, which were positively related to the NTU‐COF thickness. Correspondingly, the contact angle on the NTU‐COF was 139.3° (Figure 5c). The phenomenon was believed to be due to the micro‐nano flake structure of the shell and the aromatic ring skeleton.^41^, ^42^

**Figure 6:**
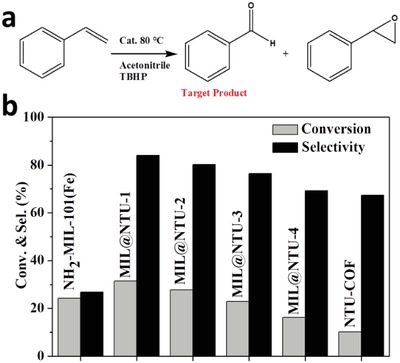
a) Styrene oxidation using *tert*‐butyl hydroperoxide (TBHP) as the oxidant. b) Styrene oxidation reaction catalyzed by different catalysts.

It was expected that the hydrophobic shell may improve catalytic efficiency significantly and porous shell could control conditions to transfer process. To demonstrate the intrinsic properties of the MOFs@COFs hybrid materials, the oxidation of styrene,^43^, ^44^ a classical and important reaction, was chosen as a probe reaction (**Figure** **6**a). Table S2: Supporting Information and Figure 6b showed the conversion and selectivity for the oxidation of styrene on NH_2_‐MIL‐101(Fe), MIL@NTU‐*x*, and NTU‐COF. NH_2_‐MIL‐101(Fe) coated with NTU‐COF exhibited much higher selectivity for target product benzaldehyde than NH_2_‐MIL‐101(Fe). The selectivity of styrene reached 84% after 12 h for the MIL@NTU‐1, about three times larger than that of the pure NH_2_‐MIL‐101(Fe) (only 26%), which indicates that the shell can significantly affect the catalytic selectivity. Remarkably, the MIL@NTU‐1 showed a slightly improved conversion (32%) compared with NH_2_‐MIL‐101(Fe) (24%), which also exhibited considerable activity compared with MOF‐based other catalysts (Table S3, Supporting Information).^45^, ^46^, ^47^, ^48^, ^49^, ^50^ While MIL@NTU‐*x* (*x* = 2–5) exhibited gradual decline in conversion but still high selectivity.

To investigate the origin of slightly increased conversion performance, the Brunauer–Emmett–Teller (BET) surface areas and pore size distribution were measured by N_2_ adsorption‐desorption isotherms (Figures S27 and S28, Supporting Information), and the results were 657, 352, 86, and 84 m^2^ g^−1^ for NH_2_‐MIL‐101(Fe), MIL@NTU‐1, MIL@NTU‐3, and NTU‐COF, and all specimens contained mesoporous channels allowing substrate and product pass through (Figure S29, Supporting Information). The decrease in the BET surface area was consistent with the result of XRD, indicating that the porous structure was destroyed gradually in the coating process. In addition, we designed a simple styrene adsorption experiment proving enrichment of the substrate around catalytic center with the COF shell (Figure 5d). Thus, these results suggest that the volcanic type conversion performance may be ascribed to the positive hydrophobicity and negative porosity loss.

**Scheme 2:**
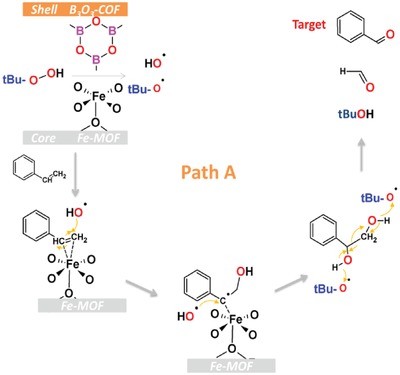
Proposed mechanism path A for styrene transformed to benzaldehyde.

To further explore the tremendous changes in selectivity, first, we should understand the active sites and mechanism of the catalytic reaction. The core Fe‐MOF consists of amino‐terephthalate linkers and Fe_3_O‐ carboxylate trimers with octahedrally coordinated metal ions binding terminal water molecules (Figure S30, Supporting Information).^34^ These water molecules can be removed, thus providing catalytically active coordinatively unsaturated sites (CUSs). As reported by previous works, the formation of target product benzaldehyde takes place via two distinct pathways using heterogeneous Lews‐acid as a catalyst (**Scheme** **2** and Figure S31: Supporting Information).^51^, ^52^ In path A, the CUSs plays as active sites for formation of hydroxyl (OH.) and *tert*‐butylhydroxy (tBuO.) radicals from *tert*‐butyl hydroperoxide (TBHP).^53^, ^54^, ^55^ The direct attack of radicals to C=C bond of styrene yields both benzaldehyde and formaldehyde without styrene epoxide. Alternatively, styrene first reacts with TBHP molecule to form styrene oxide, which further transform to benzaldehyde upon nucleophilic attack by another molecule of TBHP, as shown in path B (Figure S31, Supporting Information). We can find that styrene oxide is produced only by path B. According the products, the mechanism should include path B for MIL@NTU‐*x* catalysts. Further, as a control experiment, when replacing the substrate styrene with styrene oxide, MIL@NTU‐*x* and NTU‐COF have almost no catalytic activity for styrene oxide, while NH_2_‐MIL‐101(Fe) showed the highest conversion (15%) for styrene oxide transformed to oxidation products benzaldehyde and phenylacetaldehyde (Table S4, Supporting Information). This indicates that COF shell cooperate with MOF core promoting free radical mechanism, mainly via path A, because of B_3_O_3_ ring as Lews‐acid boosting the radicals formation. Thus, the high selectivity performance may be ascribed to the synergistic effect between COF shell and MOF core. In order to prove that the free radical mechanism is the main catalytic pathway, we took a free radical quenching experiment. Previous study showed that methanol and isopropanol were effective scavenger of hydroxyl radicals.^56^ As shown in Figure S32 in the Supporting Information, the catalytic selectivity for benzaldehyde is declined to (18%–31%) from (84%) when adding methanol and isopropanol as scavenger. Moreover the conversion also decreased, suggesting that free radical pathway was key path to form benzaldehyde for MOFs@COFs hybrid catalysts.

Furthermore, to verify the superiority of the core–shell structure, simple physical mixtures of NH_2_‐MIL‐101(Fe) and NTU‐COF, containing the equal catalytic center CUSs to MIL@NTU‐1, were tested in the same catalytic system. As a result, the physical mixture exhibited lower conversion and selectivity than the core–shell structured MIL@NTU‐1 (Figure S33 and Table S5, Supporting Information), indicating the priority of the structure for MIL@NTU. More importantly, this suggests the shell plays an important role in transfer process. MOFs unsaturated Fe^3+^ plays as active catalytic site.^18^ The COF shell gathers the hydrophobic molecules substrate and enormously promotes the conversion of styrene to benzaldehyde. To address the catalytic stability, recycling experiments were performed with the MIL@NTU‐1. After each reaction, the catalyst was centrifuged, washed with acetonitrile, dried under vacuum, and reused for the subsequent cycles. The catalyst showed no obvious changes in terms of the catalytic activity and selectivity after successive reuses of up to four cycles (Figure S34, Supporting Information). TEM and FESEM images showed that the structure of the used MIL@NTU‐1 after four cycles still maintained octahedral morphology with rough surface (Figures S35 and S36, Supporting Information), and its crystallinity still remained modest essentially just with a little decline (Figure S37, Supporting Information).

## 3 Conclusion

In conclusion, we open up a facile method for the construction of NH_2_‐MIL‐101(Fe)@NTU‐COF core–shell hybrids, which addresses the cooperation of MOFs and COFs for heterogeneous catalysis. By coating on MOFs with hydrophobic NTU‐COF shell, their pore environment and the hydrophilic‐hydrophobic properties are successfully modified. More importantly, MIL@NTU‐1 exhibited the enhanced conversion (32%) and selectivity (84%) of styrene, superior to those of 24% and 26% for the NH_2_‐MIL‐101(Fe), respectively. This is mainly ascribed to that NH_2_‐MIL‐101(Fe) provides the coordinative unsaturated catalytic sites, NTU‐COF shell gathers the hydrophobic molecules substrate and promotes the radical mechanism for styrene directly to benzaldehyde. Hence, the novel NH_2_‐MIL‐101(Fe)@NTU‐COF hybrid core–shell architectures may be competent for targeting important and challenging selective reactions. It is expected that this gentle synthetic method with effective design MOF/COF‐based hybrids will reveal new opportunities for other heterogeneous catalysts via solving the wettability problem, and realize multifunction applications, such as energy, environment, and so on.

## Conflict of Interest

The authors declare no conflict of interest.

## Supporting information

Supplementary
