# Supplementary material for: One‐Step Construction of Hydrophobic MOFs@COFs Core–Shell Composites for Heterogeneous Selective Catalysis
Source: Adv Sci (Weinh). 2019 Feb 20;6(8):1802365. doi: 10.1002/advs.201802365 (PMC6468976; doi:10.1002/advs.201802365)
Supplement: Supplementary file 1 — Supplementary [file ADVS-6-1802365-s001.pdf]

Copyright WILEY-VCH Verlag GmbH & Co. KGaA, 69469 Weinheim, Germany, 2019.

ADVANCED SCIENCE
Open Access

Supporting Information

for *Adv. Sci.,* DOI: 10.1002/advs.201802365

One-Step Construction of Hydrophobic MOFs@COFs Core–Shell Composites for Heterogeneous Selective Catalysis

*Mengke Cai, Yinle Li, Qinglin Liu, Ziqian Xue, Haiping Wang, Yanan Fan, Kelong Zhu, Zhuofeng Ke, Cheng-Yong Su, and Guangqin Li**

One step construction of hydrophobic MOFs@COFs core-shell composites for heterogeneous selective catalysis

Mengke Cai,[a] Yinle Li,[a] Qinglin Liu,[a] Ziqian Xue,[a] Haiping Wang,[a] Yanan Fan,[a] Kelong Zhu,[a] Zhuofeng Ke,[b] Cheng-Yong Su,[a] Guangqin Li[a]*

SUPPORTING INFORMATION

a MOE Laboratory of Bioinorganic and Synthetic Chemistry, Lehn Institute of Functional Materials, School of Chemistry, Sun Yat-Sen University, Guangzhou 510275, P. R. China, E-mail: liguangqin@mail.sysu.edu.cn

b Key Laboratory for Polymeric Composite and Functional Materials of Ministry of Education, School of Materials Science and Engineering, Sun Yat-Sen University, Guangzhou 510275, P. R. China

Experimental Section

Materials. All chemicals are analytical grade and used as received without further purification.

Synthesis of 1, 3, 5-tris (4-nitrophenyl) benzene 4-Nitroacetophenone (25 g), toluene (100 mL), and CF_3SO_3H (1 mL) were added to a flask equipped with a water separator and a cooling condenser. The mixture was refluxed for 48 h, during this time the formed water was eliminated as a toluene azeotrope. After cooling down to room temperature, the mixture was filtered to yield a black solid product. It was washed with DMF under refluxing and filtered. This procedure was carried out twice more, and a pale yellow solid was obtained after drying. This product is insoluble in any common solvent.

Synthesis of 1, 3, 5-tris (4-aminophenyl) benzene A suspension of 1, 3, 5-tris (4-nitrophenyl) benzene (10 g, 22.7 mmol) and Pd/C (10 wt%, 2.0 g) in ethanol (200 mL) was heated to reflux. Hydrazine hydrate (30 mL) was added dropwise, and the mixture was refluxed overnight. The hot solution was filtered through celite and left undisturbed to fully crystallize the product. The solid was filtered and washed with cold ethanol. Yield: 4.38 g (55%). ^1H NMR (400 MHz, d_6-DMSO, 298K, TMS): δ 7.9 (t, 6H, J=5.8 Hz), 6.69 (d, 6H, J=8.4 Hz), 5.22 (s, 3H) ppm.

Synthesis of NH_2-MIL-101(Fe) Typically 250.6 mg of 2-Aminoterephthalic acid and 374.0 mg of $FeCl_3.6H_2O$ were dissolved in 30 mL of DMF and stirred vigorously for 2 h. Then, the mixed solution was transferred to a 50 mL Teflon-lined autoclave for the solvothermal treatment at 120 °C for 20 h. After being cooled to room temperature, the resultant brown precipitates were separated from the reaction mixture by centrifugation, and washed thoroughly with DMF and ethanol to remove any unreacted starting materials. Finally, the obtained solid was dried in vacuum at 60 °C for 24 h for further characterization and application.

Synthesis of NTU-COF 100 mg (0.28 mmol) of 1, 3, 5-tris (4-aminophenyl)-benzene (TAPB) and 130 mg (0.87 mmol) of 4-formylphenylboronic acid (4-FPBA) were dissolved in solution of 1, 4-dioxane/mesitylene (1/1 v/v, 10 mL) in a 100mL thick walled pressure bottle. The bottle was tightened by the screw cap and then sonicated for 30min. The reaction mixture was heated at 120 °C for 3 d. The

precipitate was isolated by centrifugation and washed with anhydrous 1,4-dioxane. The resultant material was purified by Soxhlet extraction using dichloromethane, dried in vacuum at 60 °C for 12 h to afford NTU-COF as a yellowish powder.

Synthesis of MIL@NTU-1 30 mg of NH_2-MIL-101(Fe) was suspended in solution of 1, 4-dioxane/mesitylene (1/1 v/v, 10 mL) in a 100mL thick walled pressure bottle. The suspension was sonicated for 30 min. Subsequently, various amounts of TAPB and 4-FPBA were added to the suspension such as 10 mg TAPB and 13 mg 4-FPBA. The suspension was continued to sonicate for another 30 min. After that, the reaction mixture was heated at 120 °C for 3 d. The precipitate was isolated by centrifugation and washed with anhydrous 1,4-dioxane. The resultant material was purified by Soxhlet extraction using dichloromethane, dried in vacuum at 60 °C for 12 h.

Synthesis of MIL@NTU-2. The synthesis of MIL@NTU-2 is similar to that of MIL@NTU-1 but using 20 mg TAPB and 26 mg 4-FPBA.

Synthesis of MIL@NTU-3. The synthesis of MIL@NTU-3 is similar to that of MIL@NTU-1 but using 30 mg TAPB and 39 mg 4-FPBA.

Synthesis of MIL@NTU-4. The synthesis of MIL@NTU-4 is similar to that of MIL@NTU-1 but using 40 mg TAPB and 52 mg 4-FPBA.

Synthesis of MIL@NTU-5. The synthesis of MIL@NTU-5 is similar to that of MIL@NTU-1 but using 50 mg TAPB and 65 mg 4-FPBA.

Carbonization of MIL@NTU-3 and NH_2-MIL-101(Fe). Typically the MIL@NTU-3 was thermally treated at 800 °C in a tube furnace for 3 h under an Ar atmosphere. Then the sample was vacuum dried for further characterization. The NH_2-MIL-101(Fe) was treated using the same carbonization procedures.

Adsorption experiment. The styrene (10 mM) was dissolved in acetonitrile. Well-dried adsorbent (10mg) was added to the 10 mL styrene solution (10 mM in acetonitrile). After adsorption for 10 minutes, the solution was removed. The remaining powder was dissolved in 10 mL acetonitrile and then the solution was collected by centrifugation, which was then diluted 100 times. The moles of adsorption were identified and quantified using GC-MS. Based on this, the amounts of styrene absorbed within materials were calculated.

Catalytic oxidation of styrene. The oxidation of styrene was performed in a 25 mL, three-necked flask equipped with a liquid condenser. In a catalytic run, the catalyst (10 mg), styrene (2 mmol) and tert-butyl hydroperoxide (TBHP, 6 mmol) were added to 10 mL of acetonitrile (CH_3CN). Then the mixture was refluxed at 80 °C for 12 h. After the reaction, the solid catalyst was centrifuged, washed with acetonitrile and ethanol, dried in vacuum and reused without further purification. The products were identified and quantified using a gas chromatograph and argon gas as the carrier gas. Both the injector and detector temperatures were 250 °C. The reactant conversion and product for benzaldehyde were calculated as follows:

Styrene conversion (mol%)=(moles of reactant converted)/(moles of reactant infeed)*100

Product selectivity (mol%)=(moles of product for benzaldehyde)/(moles of reactant converted)*100

Characterization

Field-emission scanning electron microscopy (FESEM) images were performed on a Hitachi SU8010 scanning electron microscope at 5.0 kV. Transmission electron microscopy (TEM) images were carried out using JEOL JEM-1400 at 120 kV. High angle annular dark field scanning transmission electron microscopy (HAADF-SEM) imaging and energy-dispersive X-ray spectroscopy (EDS) elemental mapping were carried out on JEOL ARM200 at 300 kV. Powder X-ray diffraction (PXRD) patterns were recorded on a Rigaku SmartLab diffractometer with Cu Kα (λ = 1.540598 Å) radiation operating at 30 kV and 200 mA. X-ray photoelectron spectroscopy (XPS) were performed by a VG ESCALABMKII instrument. Fourier transform infrared (FT-IR) spectra were recorded on a Bruker Alpha spectrometer. The content of Fe in different samples was determined by inductively coupled plasma spectrometer (ICP, Thermo Fisher Scientific). The contact angles were conducted using DSA30 (KRUSS GmbH,

Germany). The N_2 adsorption-desorption isotherms were collected using a Quantachrome Instruments Autosorb-iQ2-MP at 77 K. The catalytic results were tested on gas chromatography-mass spectrometry (GC-MS, SHIMAZU QP2020 (EI type) Standard Quotation).

Supplementary Figures and Tables

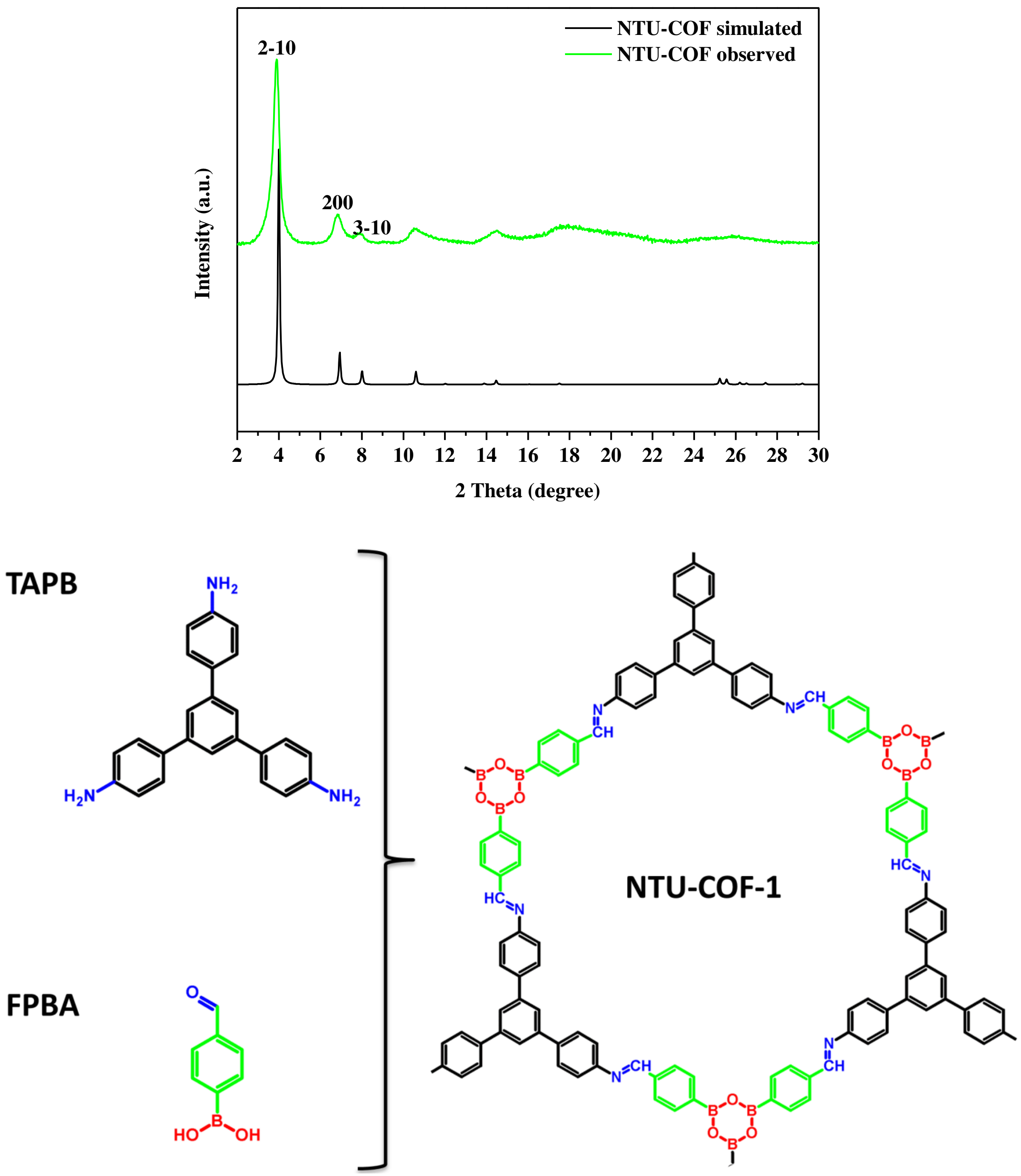

Figure S1. The PXRD patterns of observed NTU-COF (green), simulated NTU-COF (black) and corresponding molecular structure.

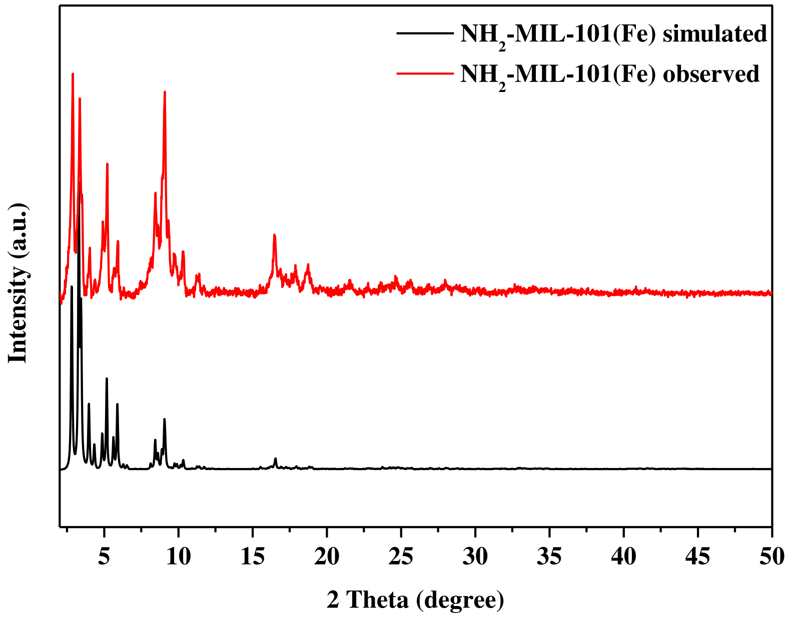

Figure S2. The PXRD patterns of observed NH_2-MIL-101(Fe) (red) and simulated NH_2-MIL-101(Fe) (black).

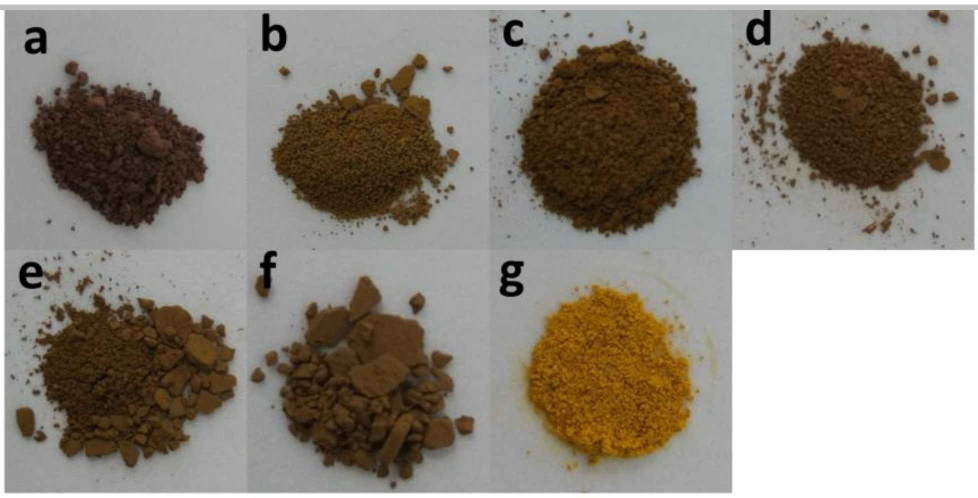

Figure S3. The optical images of a) NH_2-MIL-101(Fe); b) MIL@NTU-1; c) MIL@NTU-2; d) MIL@NTU-3; e) MIL@NTU-4; f) MIL@NTU-5; g) NTU-COF.

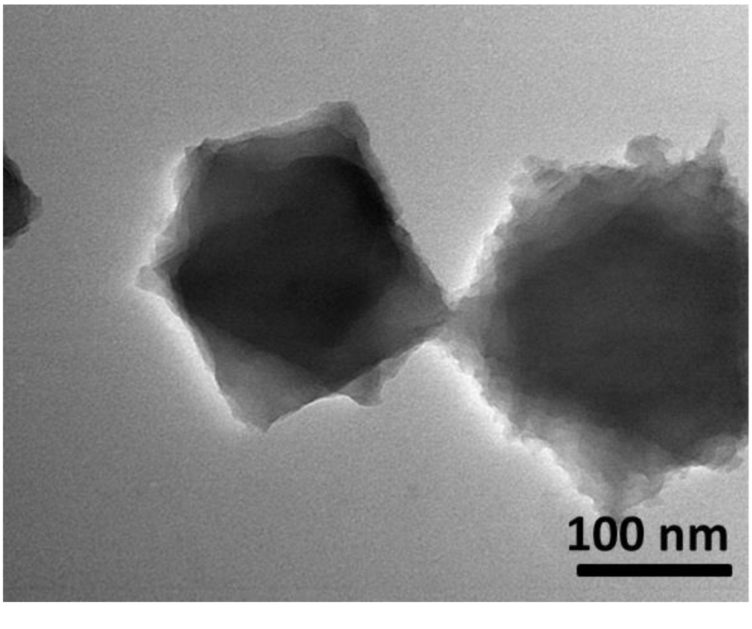

Figure S4. The TEM image of NH_2-MIL-101(Fe).

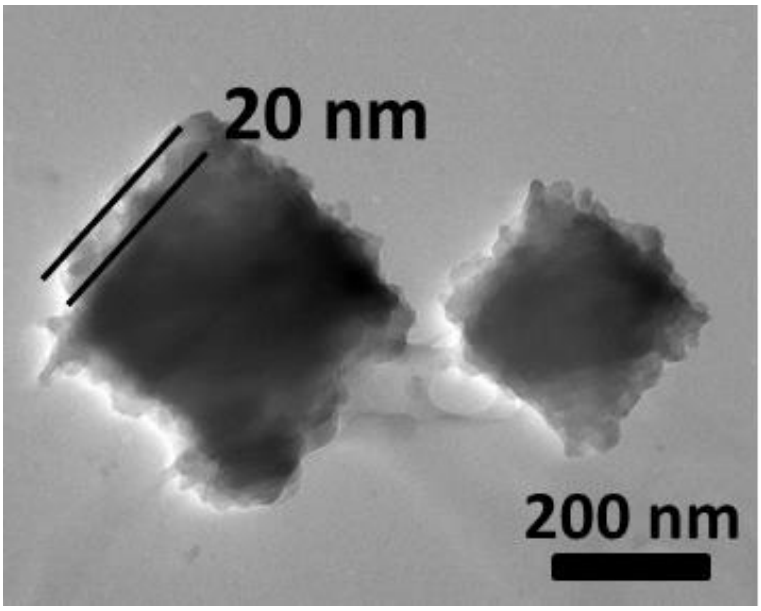

Figure S5. The TEM image of MIL@NTU-2.

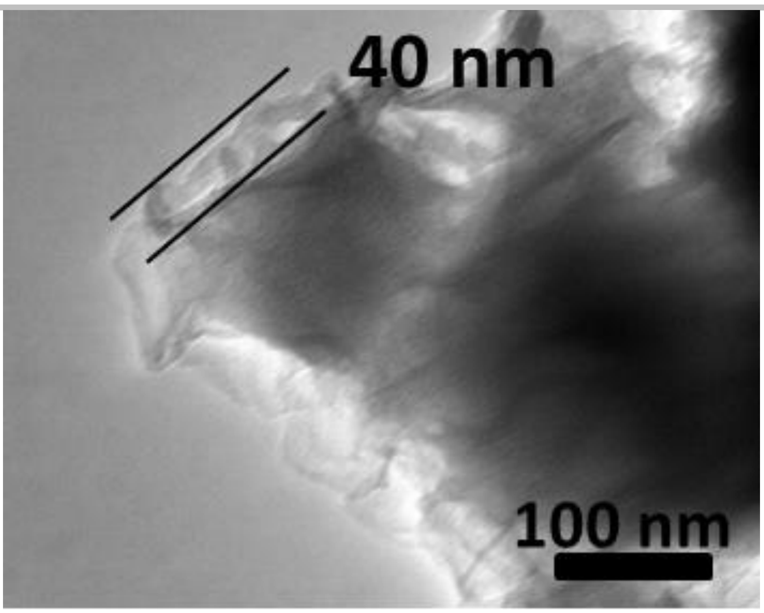

Figure S6. The TEM image of MIL@NTU-3.

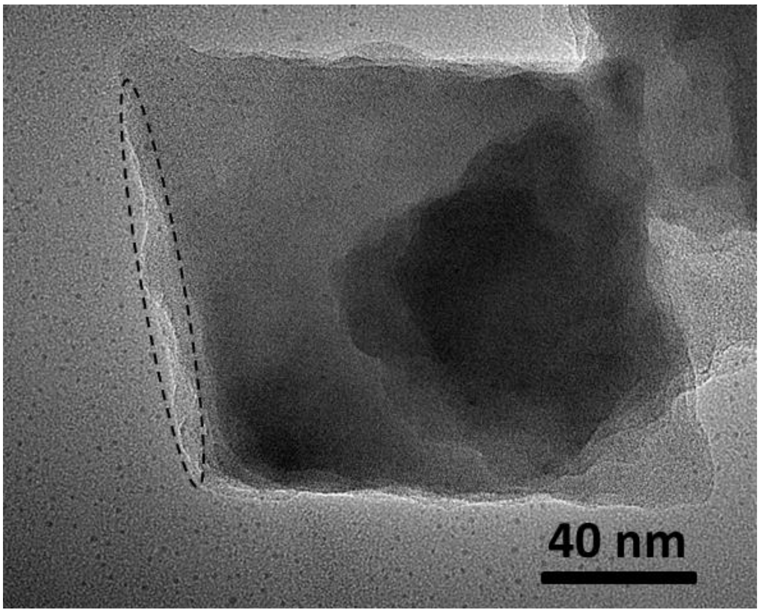

Figure S7. The TEM image of MIL@NTU-1.

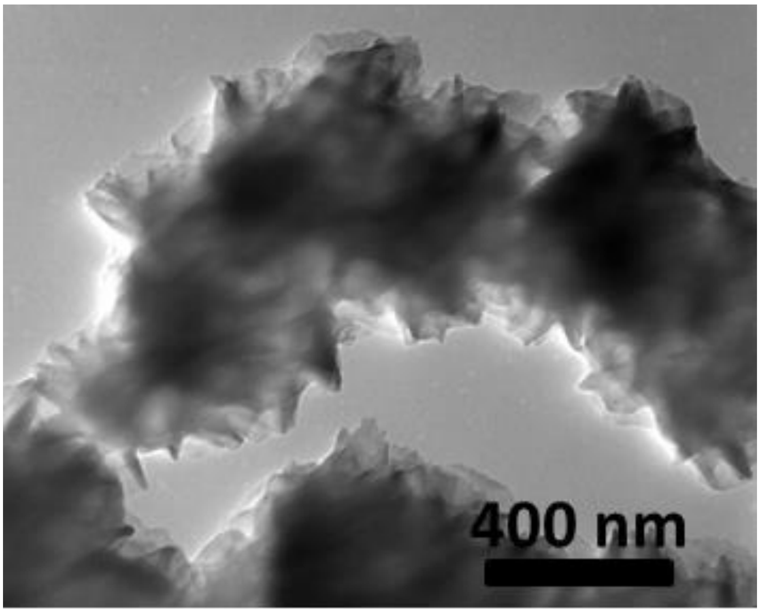

Figure S8. The TEM image of MIL@NTU-4.

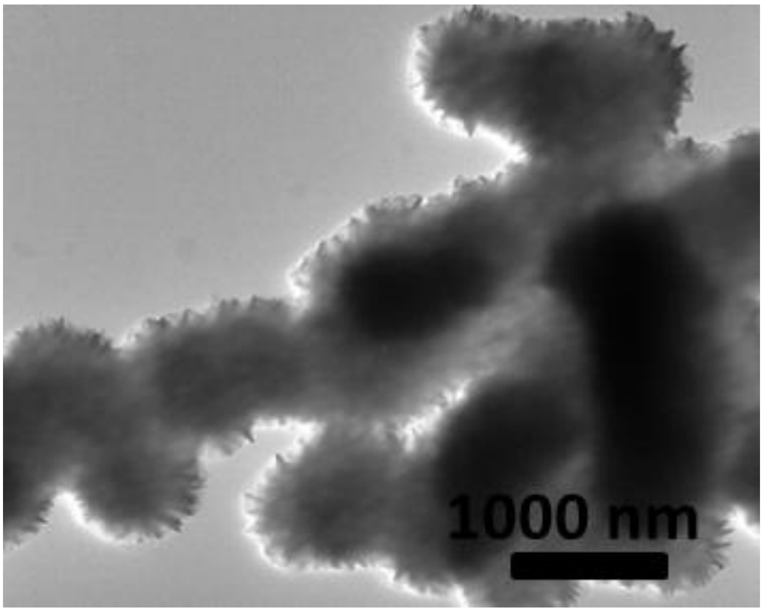

Figure S9. The TEM image of MIL@NTU-5.

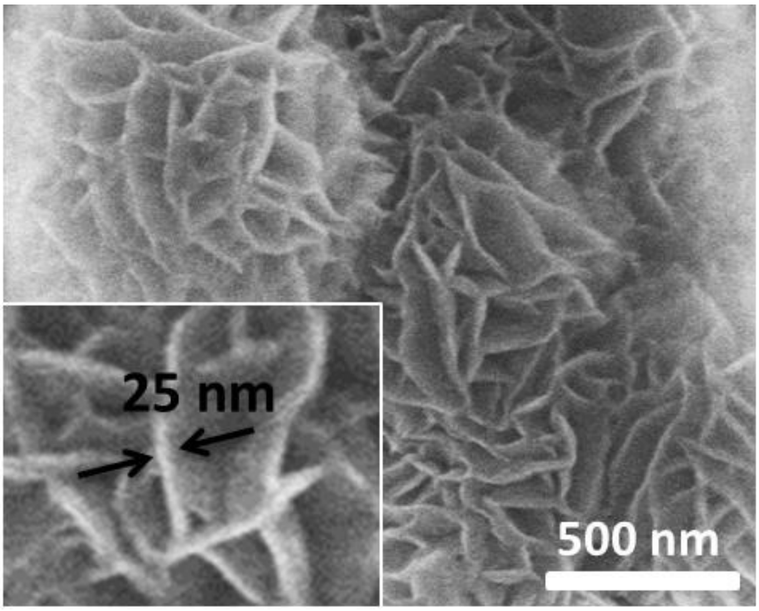

Figure S10. The SEM image of MIL@NTU-5.

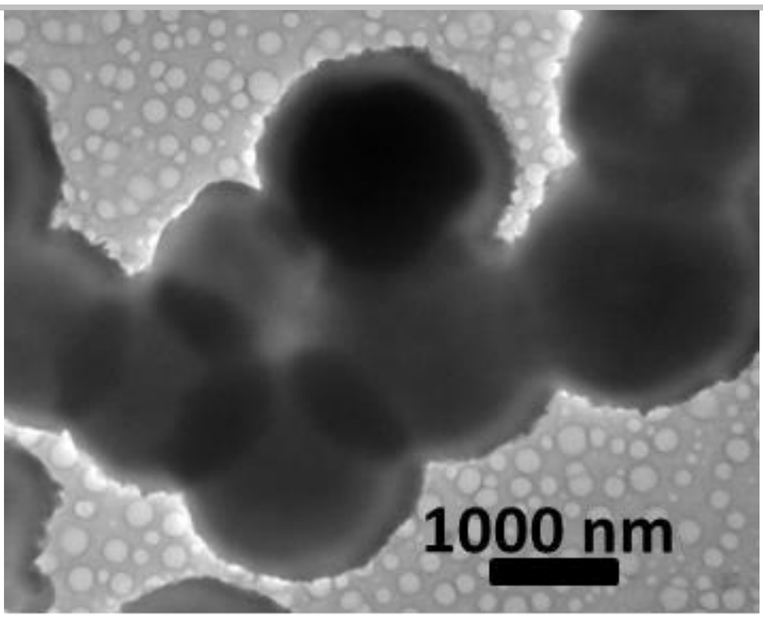

Figure S11. The TEM image of NTU-COF.

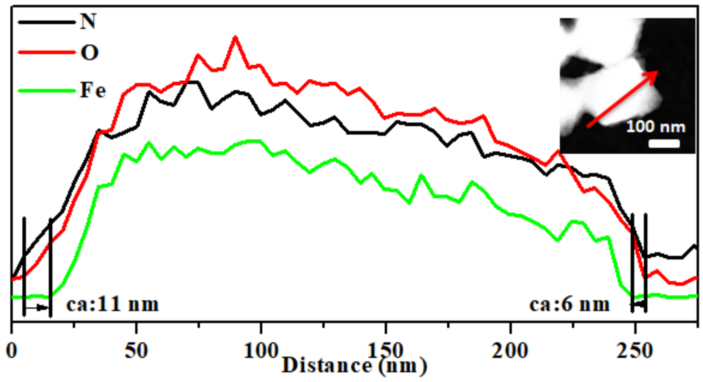

Figure S12. EDX lines scan profile of N, O and Fe elements as indicated by the red line in inset figure.

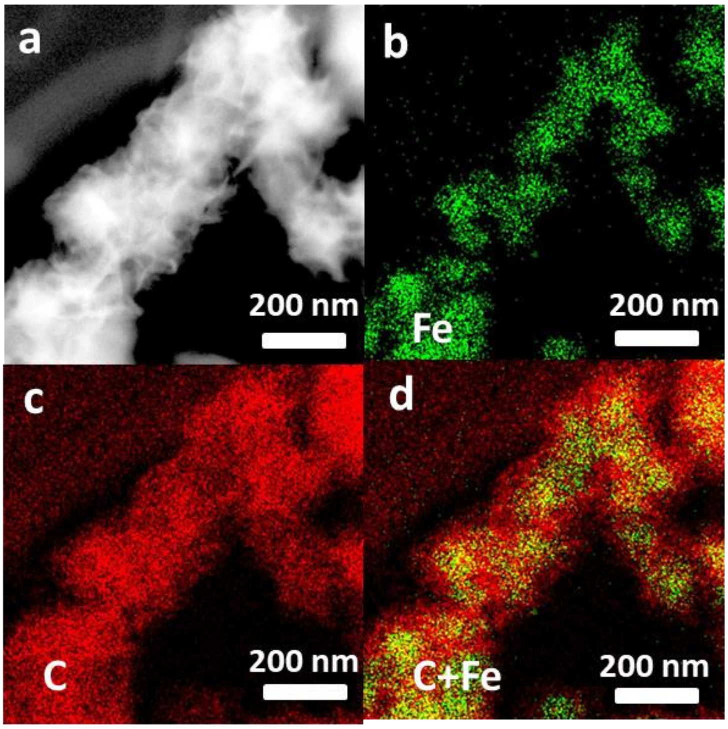

Figure S13. a) HAADF-STEM image of MIL@NTU-3. Elemental mapping images of Fe (b), C (c), C and Fe (d) of the core-shell structure shown in (a).

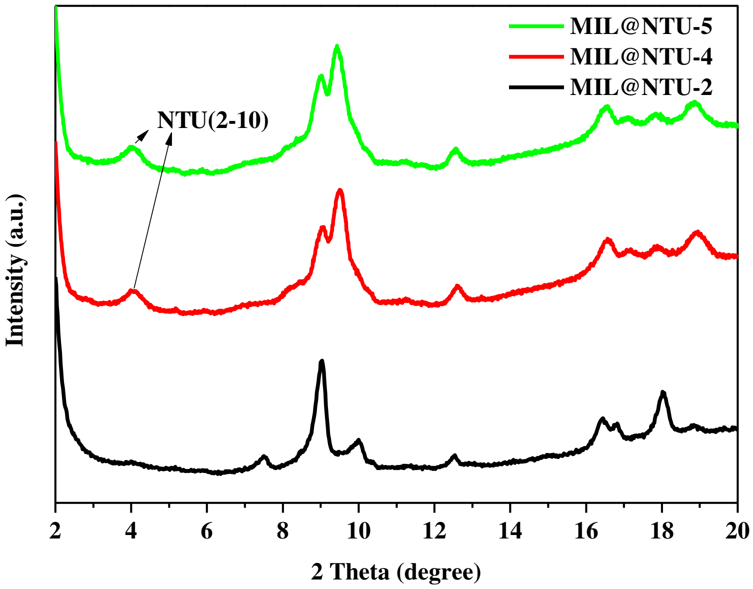

Figure S14. The PXRD patterns of MIL@NTU-2 (black), MIL@NTU-4 (red) and MIL@NTU-5 (green).

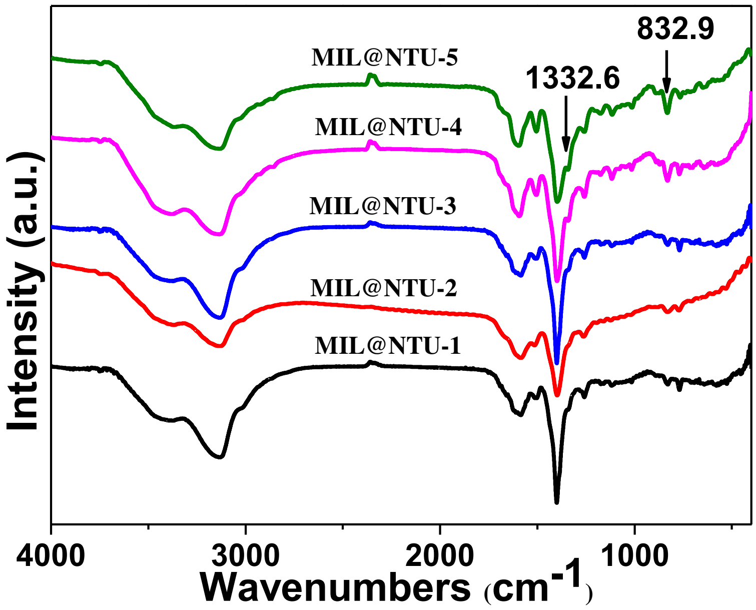

Figure S15. FT-IR spectra of a) MIL@NTU-1; b) MIL@NTU-2; c) MIL@NTU-3; d) MIL@NTU-4; e) MIL@NTU-5.

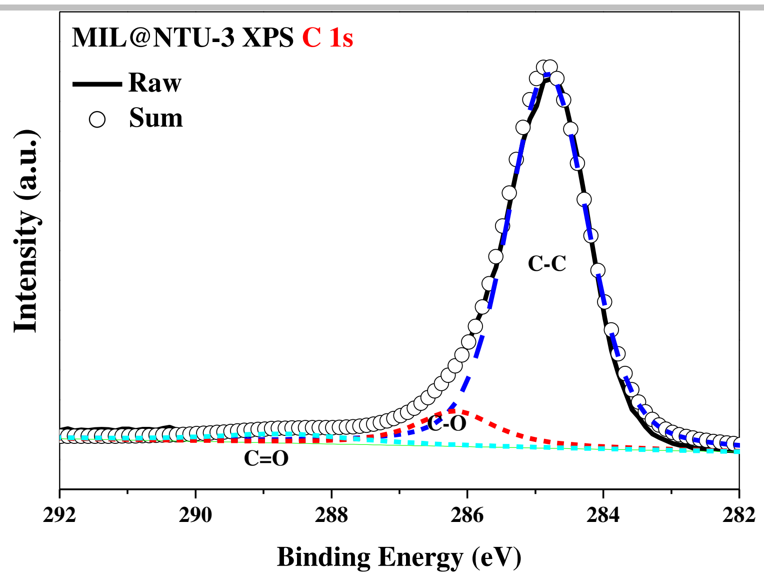

Figure S16. C 1s XPS of MIL@NTU-3.

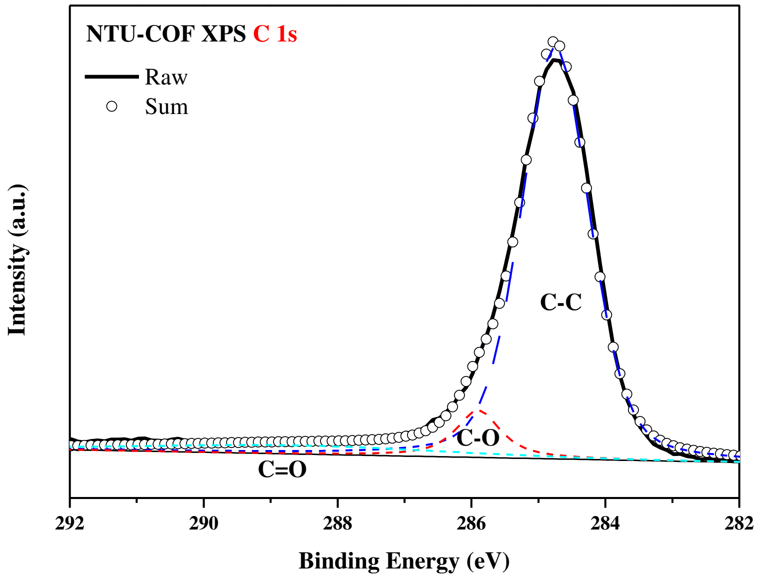

Figure S17. C 1s XPS of NTU-COF.

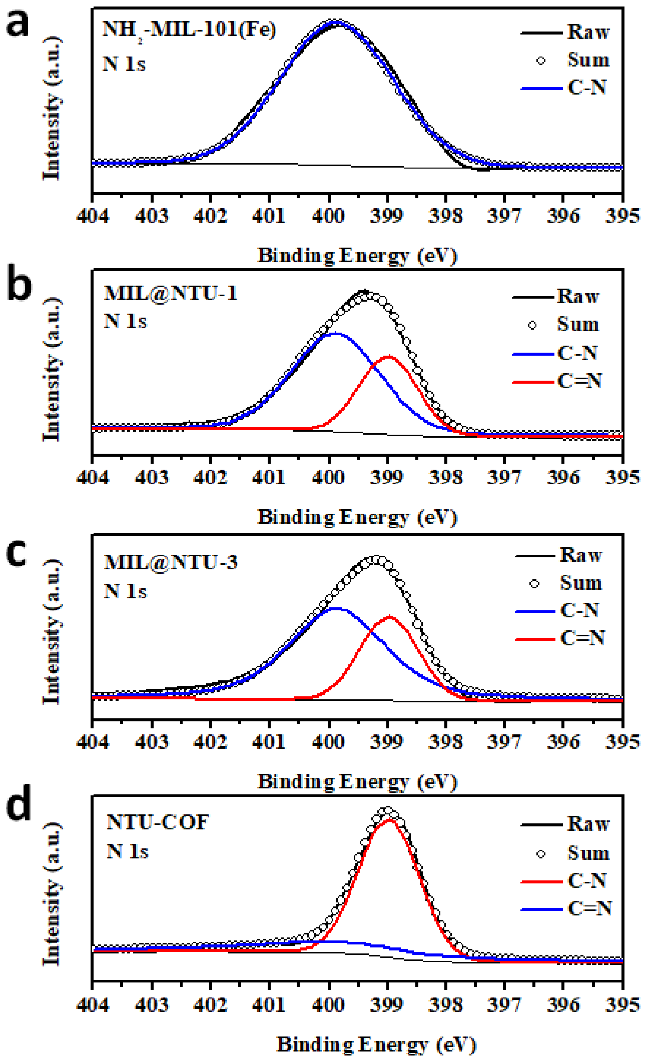

e

Binding Energy/eV	C-N	C=N
NH2-MIL-101(Fe)	399.9	/
MIL@NTU -1	399.9	398.9
MIL@NTU -3	399.9	398.9
NTU-COF	399.9	398.9

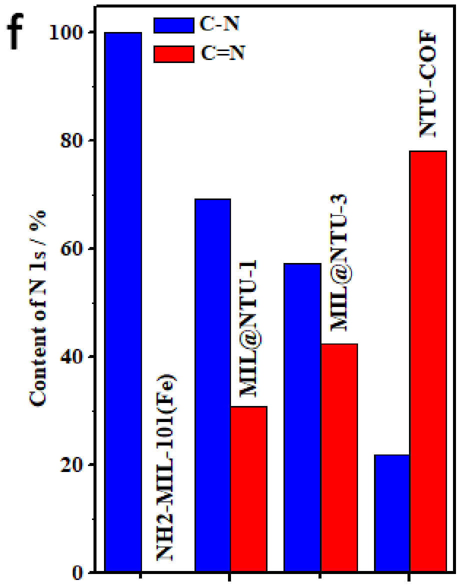

Figure S18. XPS N 1s spectrum of a) NH_2-MIL-101(Fe); b) MIL@NTU-1; c) MIL@NTU-3; d) NTU-COF. e) The position of C-N and C=N binding energy in each samples. f) Content of N 1s for C-N and C=N in each samples.

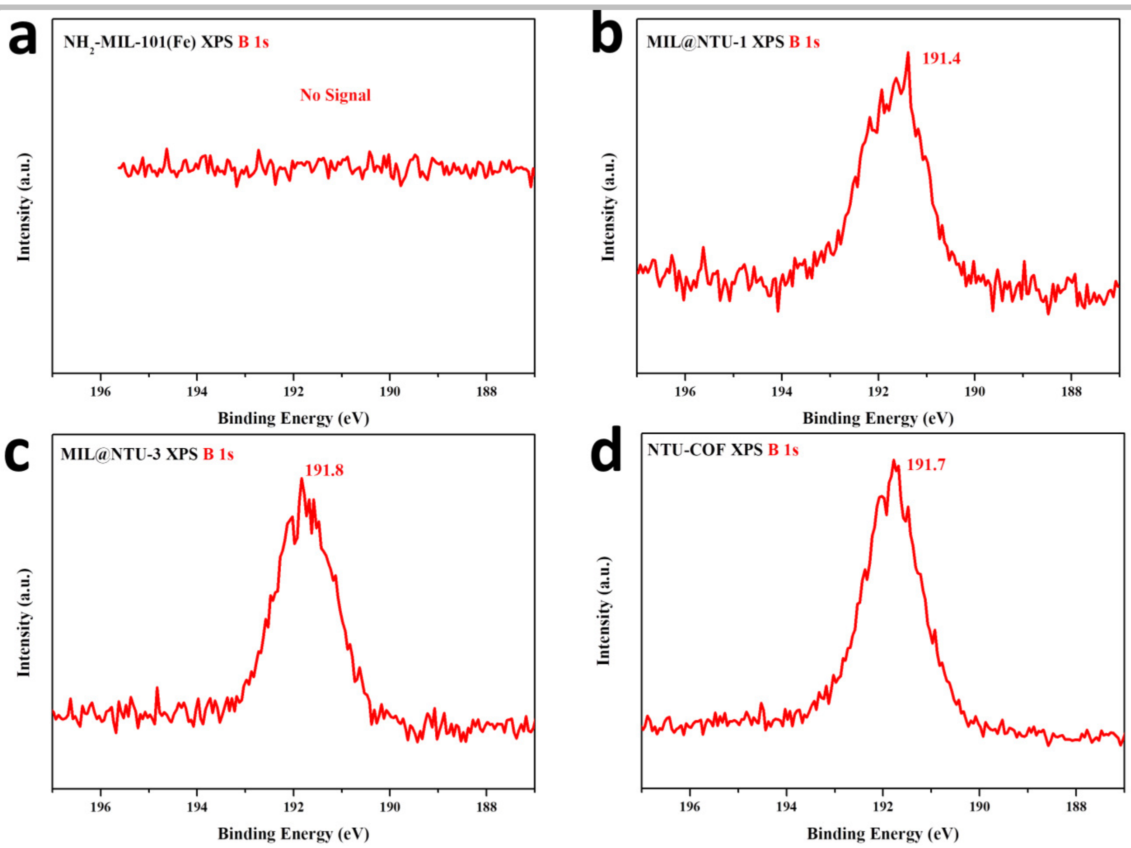

Figure S19. B 1s XPS of a) NH_2-MIL-101(Fe); b) MIL@NTU-1; c) MIL@NTU-3; d) NTU-COF.

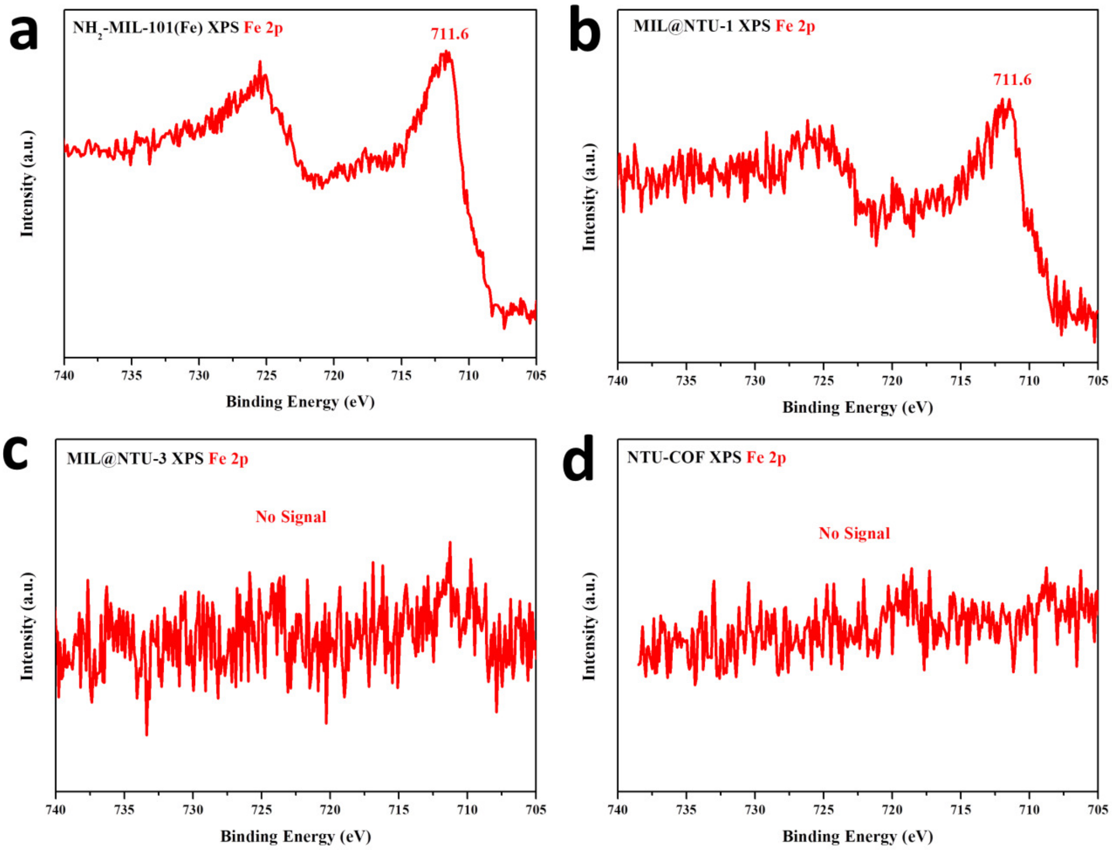

Figure S20. Fe 2p XPS of a) NH_2-MIL-101(Fe); b) MIL@NTU-1; c) MIL@NTU-3; d) NTU-COF.

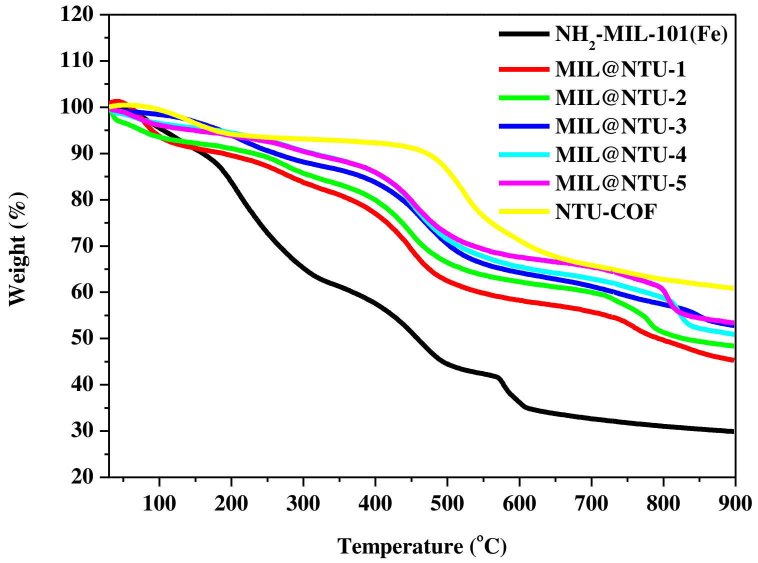

Figure S21. TGA profiles of NH_2-MIL-101(Fe), MIL@NTU-1, MIL@NTU-2, MIL@NTU-3, MIL@NTU-4, MIL@NTU-5 and MIL@NTU.

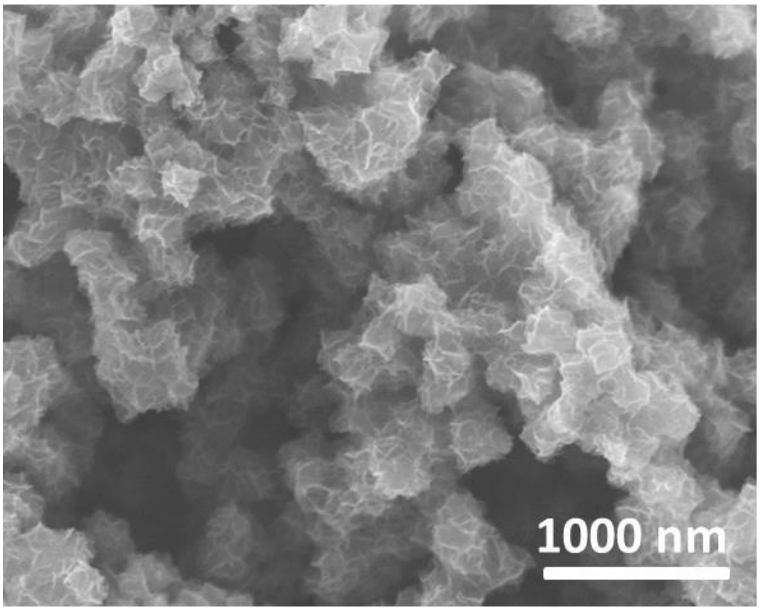

Figure S22. The FESEM image of MIL@NTU-3 after carbonization.

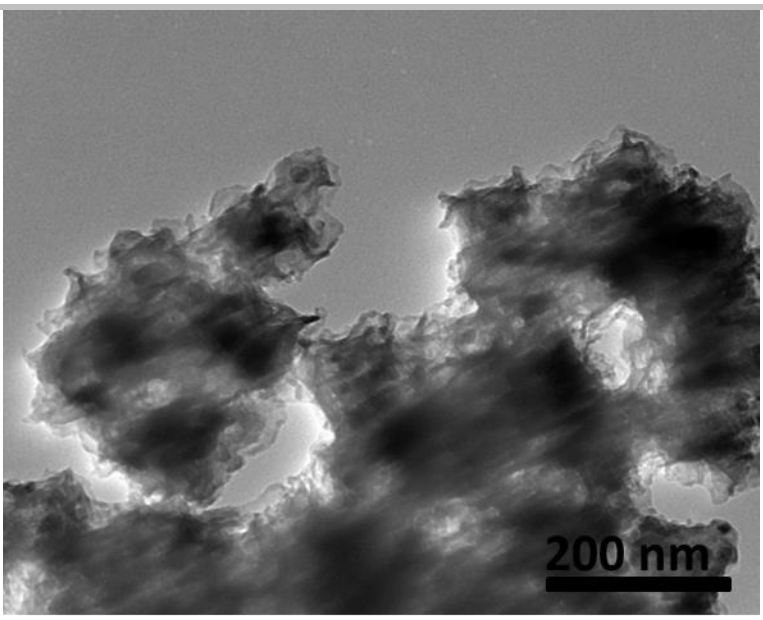

Figure S23. The TEM image of MIL@NTU-3 after carbonization.

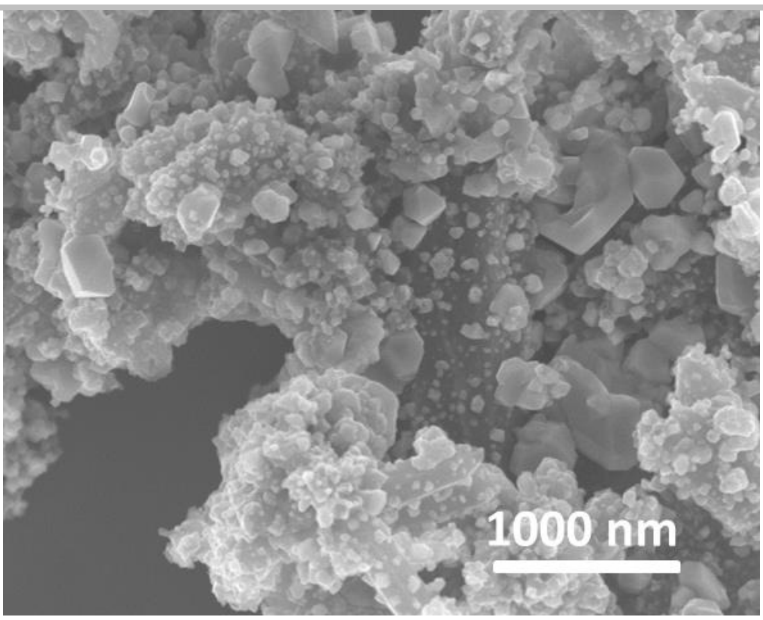

Figure S24. The FESEM image of NH_2-MIL-101(Fe) after carbonization.

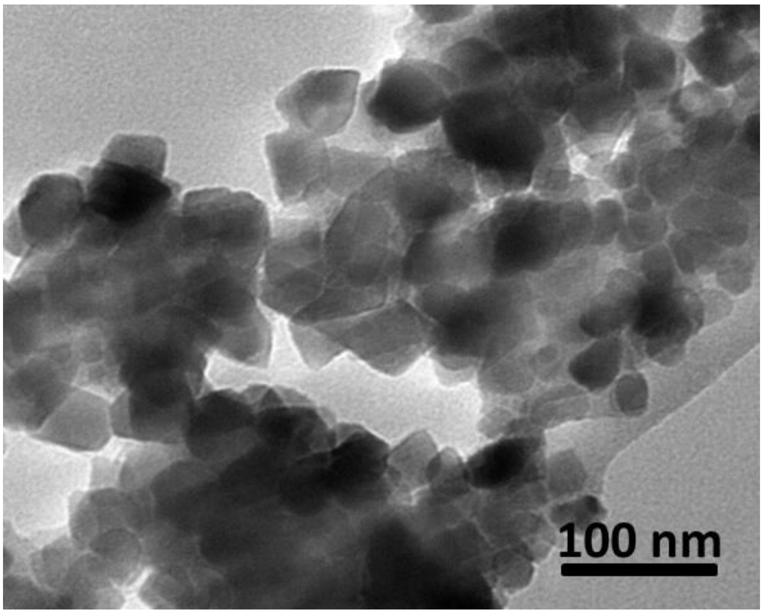

Figure S25. The TEM image of NH_2-MIL-101(Fe) after carbonization.

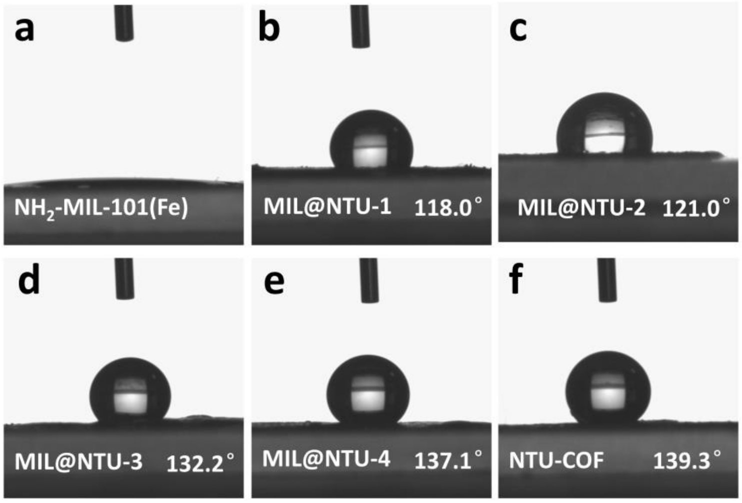

Figure S26. Water contact angle measurements of (a) NH2-MIL-101(Fe); (b) MIL@NTU-1; (c) MIL@NTU-2; (d) MIL@NTU-3; (e)MIL@NTU-4; (f) NTU-COF.

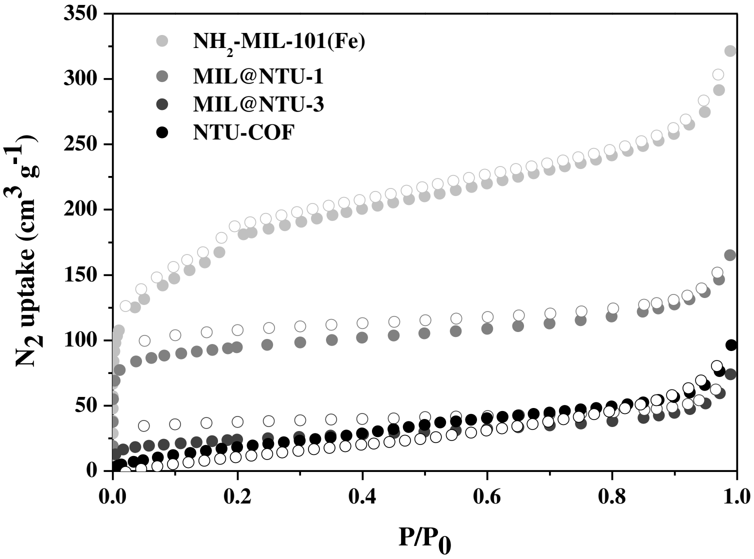

Figure S27. N_2 adsorption and desorption isotherms at 77 K of NH_2-MIL-101(Fe), MIL@NTU-1, MIL@NTU-3 and NTU-COF.

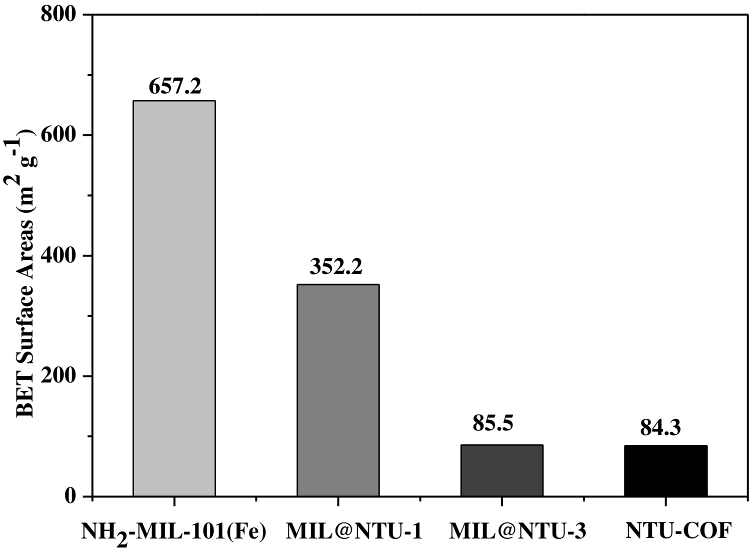

Figure S28. BET surface areas of NH_2-MIL-101(Fe), MIL@NTU-1, MIL@NTU-3 and NTU-COF.

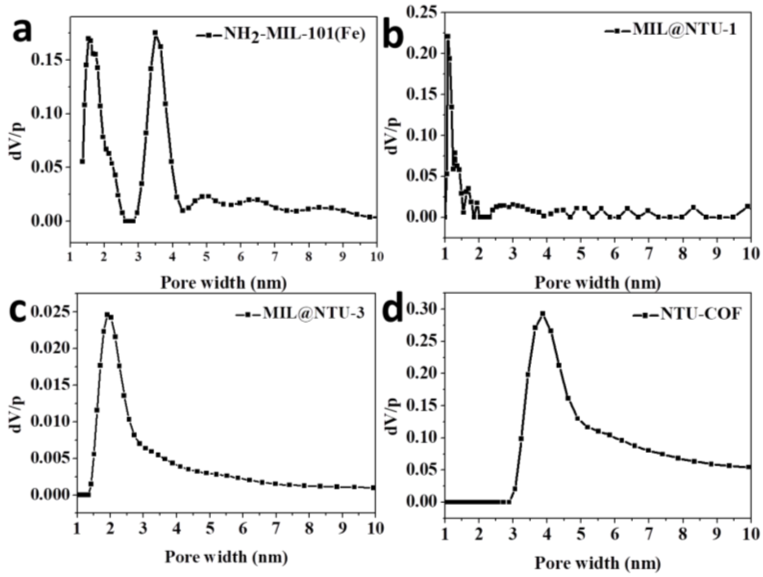

Figure S29. Pore size distribution of COFs was calculated by nonlocal density functional theory (NLDFT): a) NH_2-MIL-101(Fe); b) MIL@NTU-1; c) MIL@NTU-3; d) NTU-COF.

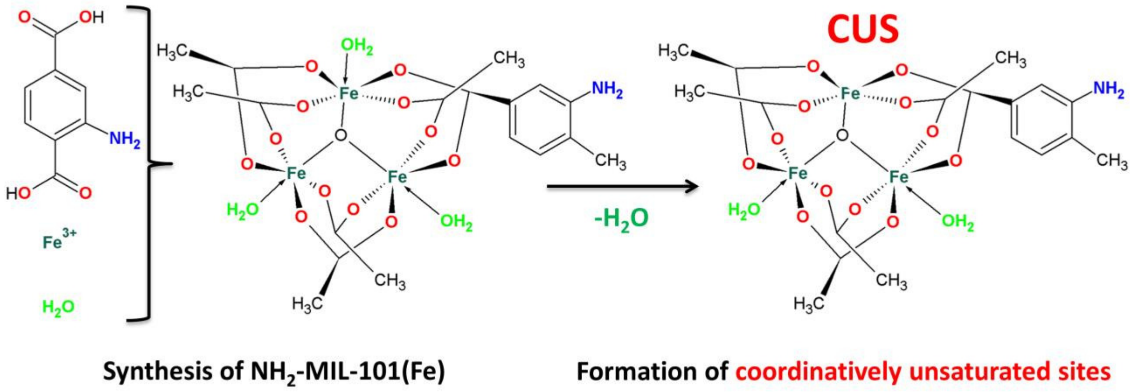

Figure S30. Formation of coordinatively unsaturated sites in NH_2-MIL-101(Fe).

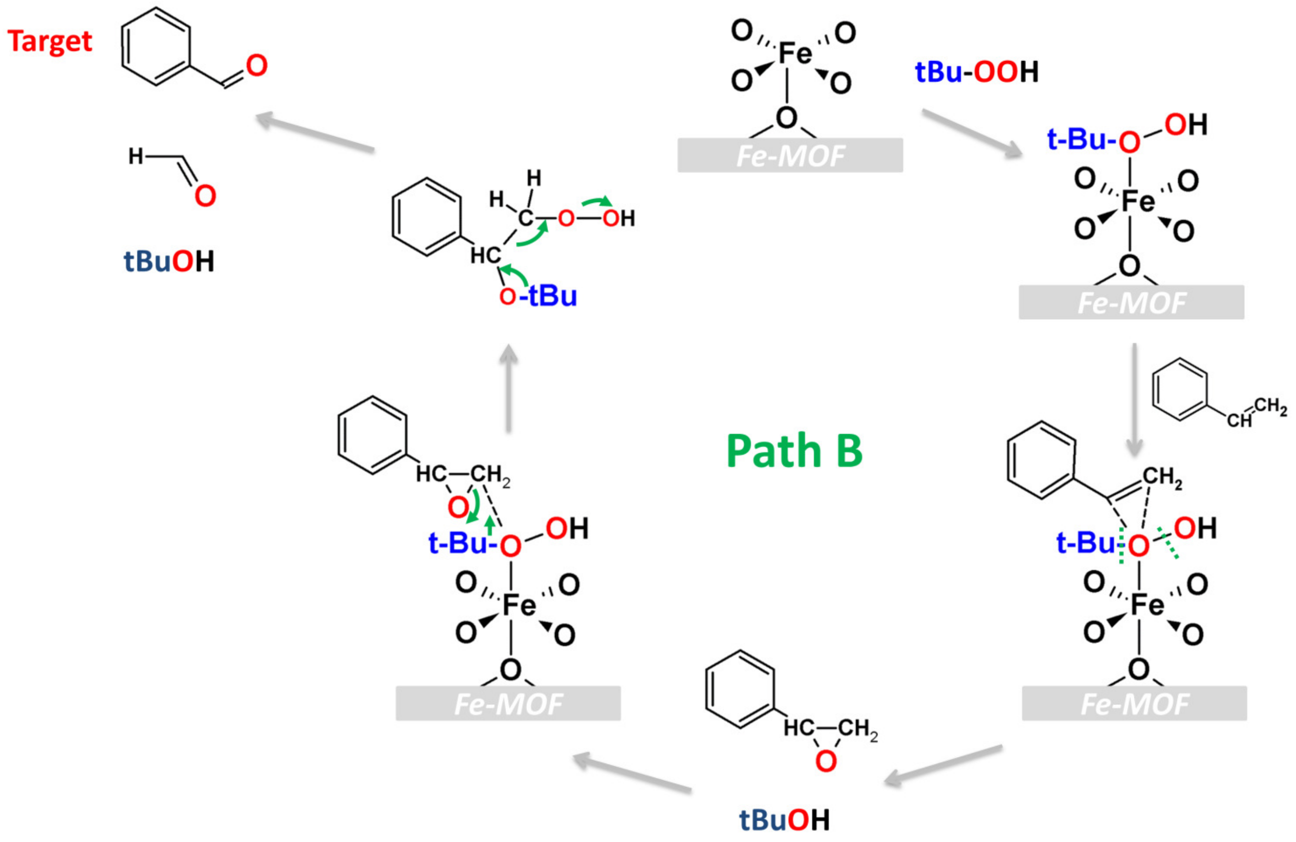

Figure S31. Proposed mechanism path B for styrene transform to benzaldehyde indirectly.

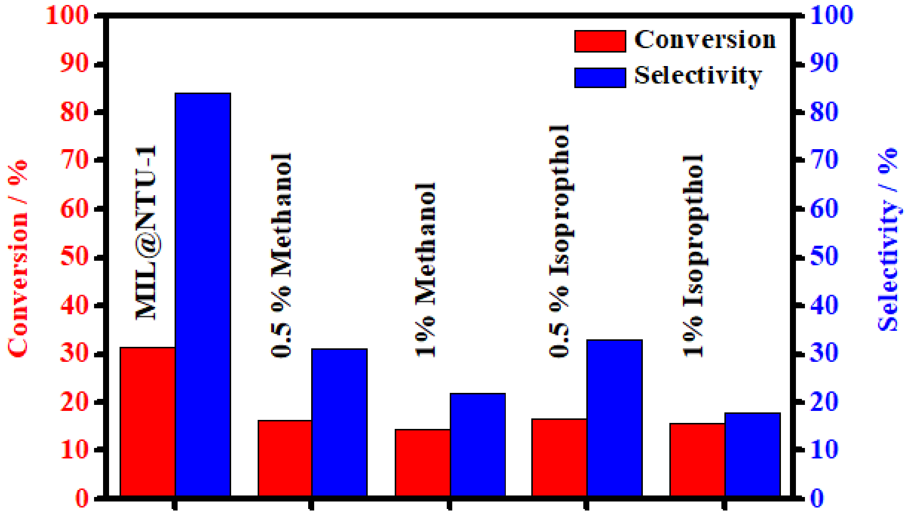

Figure S32. Changes in conversion and selectivity in the presence of methanol and isopropanol. The percentage of additional quencher is the mole number of the substrate. Reaction conditions: catalyst 10 mg, styrene (2 mmol), methanol/ isopropanol (5~10 umol) and TBHP (6 mmol) in acetonitrile (10 mL) at 80ºC, 12 h.

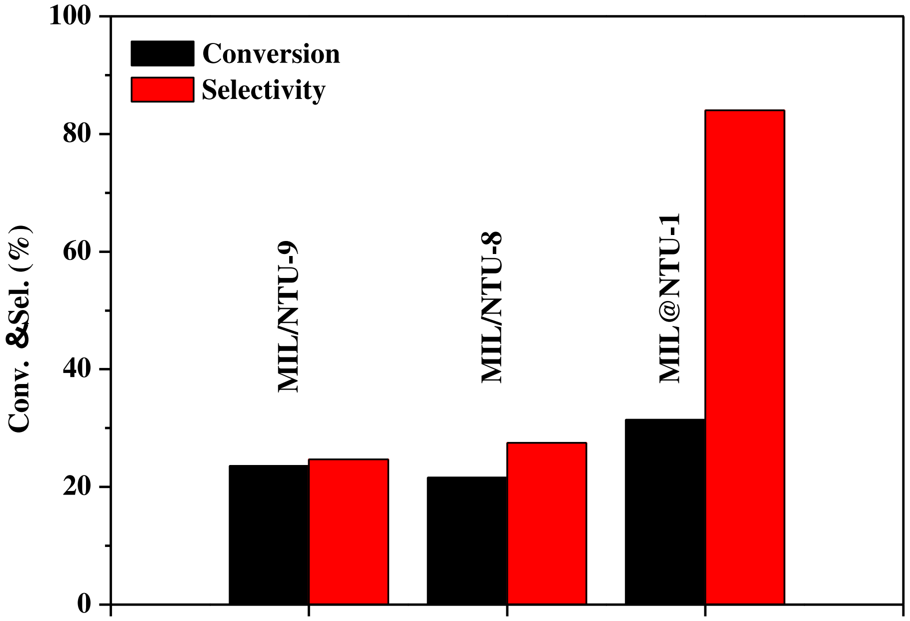

Figure S33. Styrene oxidation reaction catalyzed by simple physical mixtures (MIL/NTU-9 and MIL/NTU-8) and core-shell structure (MIL@NTU-1).

According to the Fe weight percent measured by ICP-MS (Table S4), MIL@NTU-1 could be regarded between MIL/NTU-9 and MIL/NTU-8. As expected, MIL/NTU-9 only exhibited 24% conversion, 25% selectivity and MIL/NTU-8 exhibited 22% conversion, 28% selectivity for benzaldehyde (Figure S30), which were both worse than the core-shell structure MIL@NTU-1.

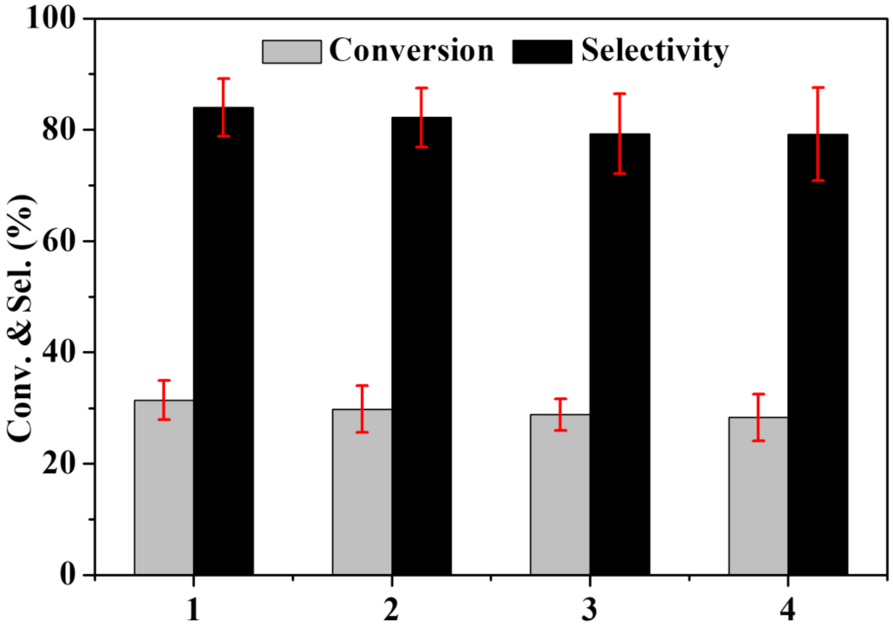

Figure S34. The recycling experiments four cycles for MIL@NTU-1.

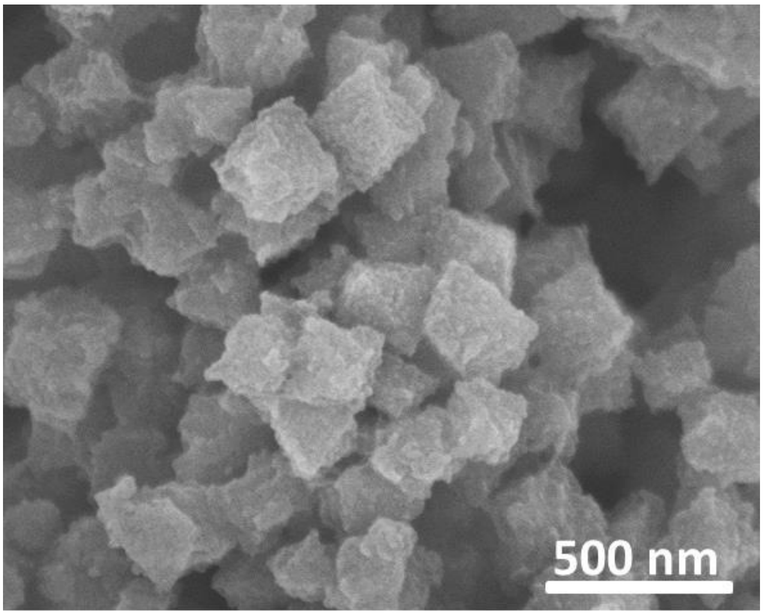

Figure S35. The FESEM image of MIL@NTU-1 after four cycles.

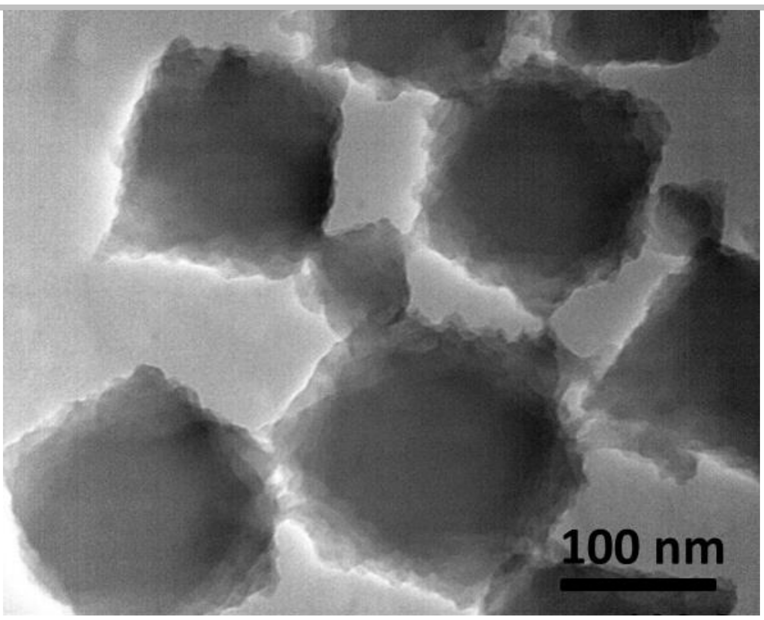

Figure S36. The TEM image of MIL@NTU-1 after four cycles.

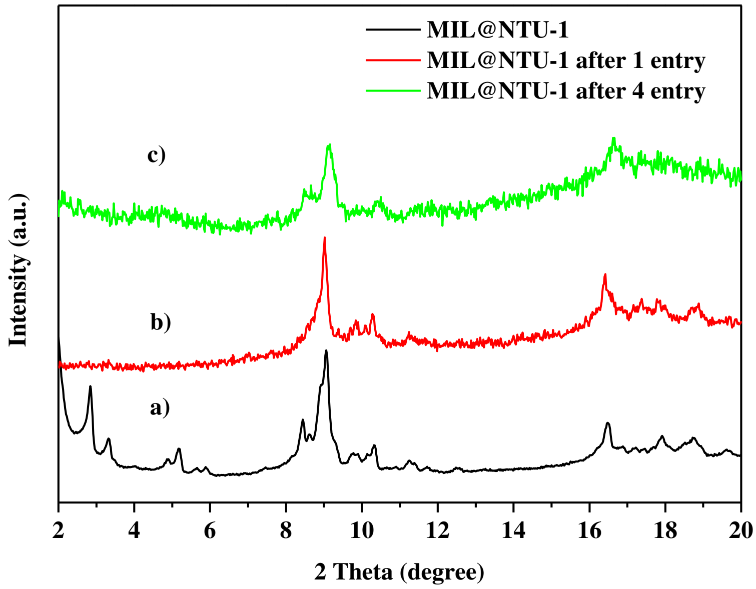

Figure S37. The PXRD patterns of MIL@NTU-1 after four cycles: a) MIL@NTU-1, b) MIL@NTU-1 after 1 entry and c) MIL@NTU-1 after 4 entry.

Table S1. Elemental atomic ratios of different samples calculated from XPS survey spectra.

Sample	Fe/ atomic %	B/ atomic %	C/ atomic %	N/ atomic %	O/ atomic %
NH_2-MIL-101(Fe)	**3.82**	**0**	62.66	7.80	25.72
MIL@NTU-1	**1.37**	**2.95**	75.21	6.29	14.18
MIL@NTU-3	**0.45**	**3.85**	80.61	6.43	8.67
NTU-COF	**0**	**5.64**	80.67	5.36	8.33

Table S2. Selective oxidation of styrene by different catalysts.[a]

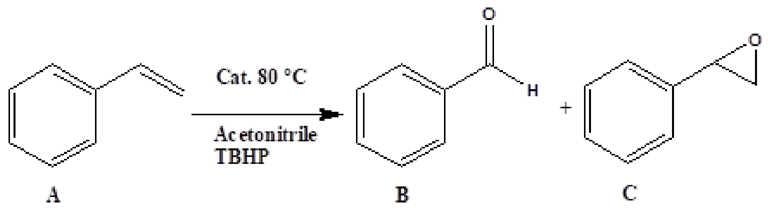

Entry	Catalysts	Conv. [%][b]	Selectivity[%] [b] B	C
1	Blank[c]	9.79	12.4	87.6
2	NH_2-MIL-101(Fe)	24.3	26.8	72.2
3	NTU-COF	10.2	67.4	32.6
4	MIL@NTU-1	31.4	84.0	16.0
5	MIL@NTU-2	27.8	80.2	19.8
6	MIL@NTU-3	22.9	76.5	23.5
7	MIL@NTU-4	16.3	69.3	30.7
8	MIL@NTU-5	10.8	65.7	34.3

[a] Reaction conditions: catalyst 10 mg, styrene (2 mmol), and TBHP (6 mmol) in acetonitrile (10 mL) at 80°C, 12 h.

[b] Determined by GC-MS.

[c] Blank means no catalyst was used.

Table S3. Comparison of the activities of MOF-based and other catalysts for the oxidation of styrene.

catalysts	reaction time/ temperature	styrene conv. (%)	benzaldehyde selectivity (%)	ref.
NH_2-MIL-101(Fe)	12h / 80℃	24	27	this work
MIL@NTU-1	12h / 80℃	**32**	**84**	**this work**
MIL-101(Cr)	12h / 80℃	18	22	1
$Mo(HSY)_2$	6h / 80℃	29	32	2
Mo-COF	6h / 80℃	>99	29	2
Co_3O_4	12h / 80℃	75	66	3
$Co_{3-X}Fe_XO_4$	12h / 80℃	92	65	3
Fe_2O_3 NP	24h / 90℃	49	81	4
Fe_3O_4 NP	24h / 90℃	53	78	4
MOF-74(Cu)	20h / 80℃	0.6	100	5
MOF-74(Co)	20h / 80℃	47	35	5
Au/LDH	8h / 80℃	41	9	6
Au/TiO_2	8h / 80℃	61	10	6
Ag/UIO-66	24h / 80℃	51	39	7
Ag/UIO-66	24h / 60℃	14	26	7
Ag/UIO-66	24h / 40℃	13	54	7

Table S4. Selective oxidation of styrene oxide by different catalysts.[a]

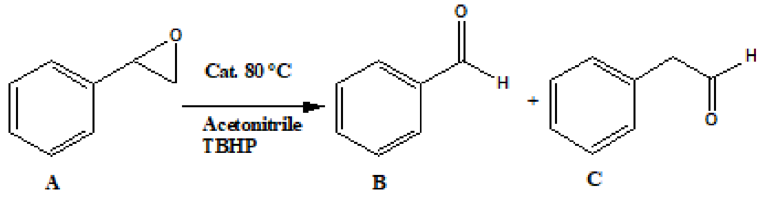

Entry	Catalysts	Conv. [%][b]	Selectivity[%] [b] B	C
1	Blank[c]	0	0	0
2	NH_2-MIL-101(Fe)	14.9	63.7	36.3
3	NTU-COF-1	0.64	37.5	62.5
4	MIL@NTU-1	2.30	62.2	37.8
5	MIL@NTU-2	2.23	59.6	40.4
6	MIL@NTU-3	1.32	45.1	54.9
7	MIL@NTU-4	1.47	48.2	51.8

8	MIL@NTU-5	2.13	53.6	46.4

[a] Reaction conditions: catalyst 10 mg, styrene oxide (2 mmol), and TBHP (6 mmol) in acetonitrile (10 mL) at 80°C, 12 h.

[b] Determined by GC-MS.

[c] Blank means no catalyst was used.

Table S5. Fe weight percent of different catalysts measured by ICP-MS.

Entry	Catalyst	Fe (mg/g)
1	NH_2-MIL-101(Fe)	146
2	MIL@NTU-1	130
3	MIL@NTU-3	103
4	MIL@NTU-5	79
5	NTU-COF	0

References

1 W. Liu, J. Huang, Q. Yang, S. Wang, X. Sun, W. Zhang, J. Liu and F. Huo, *Angew. Chem., Int. Ed.*, **2017**, *56*, 5512-5516.

2 W. Zhang, P. Jiang, Y. Wang, J. Zhang, Y. Gao and P. Zhang, *RSC Adv.*, **2014**, *4*, 51544-51547.

3 J. Sun, Y. Li, X. Liu, Q. Yang, J. Liu, X. Sun, D. G. Evans and X. Duan, *Chem. Commun.*, **2012**, *48*, 3379-3381.

4 M. J. Rak, M. Lerro and A. Moores, *Chem. Commun.*, **2014**, *50*, 12482-12485.

5 Y. Fu, L. Xu, H. Shen, H. Yang, F. Zhang, W. Zhu and M. Fan, *Chem. Eng. J.*, **2016**, *299*, 135-141.

6 F. Zhang, X. Zhao, C. Feng, B. Li, T. Chen, W. Lu, X. Lei and S. Xu, *ACS Catal.*, **2011**, *1*, 232-237.

7 Y.-X. Li, Z.-Y. Wei, L. Liu, M.-L. Gao and Z.-B. Han, Inorg. *Chem. Commun.*, **2018**, *88*, 47-50.
